# Fundamentals and Advances in Stimuli-Responsive Hydrogels and Their Applications: A Review

**DOI:** 10.3390/gels11010030

**Published:** 2025-01-02

**Authors:** Iryna S. Protsak, Yevhenii M. Morozov

**Affiliations:** 1 Department of Functional Materials and Catalysis, University of Vienna, Währinger Strasse 42, 1090 Vienna, Austria; iryna.protsak@univie.ac.at; 2 AIT-Austrian Institute of Technology, Giefinggasse 4, 1210 Vienna, Austria

**Keywords:** hydrogels, stimuli-responsive hydrogels, mechanical properties, swelling kinetics, biocompatibility, biodegradability, structure engineering, applications, current challenges, future prospects

## Abstract

This review summarizes the fundamental concepts, recent advancements, and emerging trends in the field of stimuli-responsive hydrogels. While numerous reviews exist on this topic, the field continues to evolve dynamically, and certain research directions are often overlooked. To address this, we classify stimuli-responsive hydrogels based on their response mechanisms and provide an in-depth discussion of key properties and mechanisms, including swelling kinetics, mechanical properties, and biocompatibility/biodegradability. We then explore hydrogel design, synthesis, and structural engineering, followed by an overview of applications that are relatively well established from a scientific perspective, including biomedical uses (biosensing, drug delivery, wound healing, and tissue engineering), environmental applications (heavy metal and phosphate removal from the environment and polluted water), and soft robotics and actuation. Additionally, we highlight emerging and unconventional applications such as local micro-thermometers and cell mechanotransduction. This review concludes with a discussion of current challenges and future prospects in the field, aiming to inspire further innovations and advancements in stimuli-responsive hydrogel research and applications to bring them closer to the societal needs.

## 1. Introduction

Hydrogels are a type of soft matter formed when a crosslinked, specific polymer network comes into contact with water or biological fluid. The capacity of hydrogels to absorb and retain large amounts of water stems from hydrophilic groups bonded to the polymer structure, while crosslinks between the chains in the network provide resistance to dissolution [1]. Since hydrogels are a form of soft matter, it is helpful to first briefly clarify what soft matter is and what makes it so intriguing for applications before describing the characteristics and applications of hydrogels in detail. In the approximation of small deformations, Young’s modulus, which typically characterizes the elasticity of hydrogels, scales as *E*/*l*^3^, where *E* represents a typical energy, and *l* is a characteristic length scale of the hydrogel system in question. By definition, soft matter easily distorts in response to external forces, meaning hydrogels must be characterized by either a small energy scale or a large length scale. In comparison to solid-state matter, where a typical energy scale corresponds to a bonding energy of approximately 40 *k*_B_*T* (with *k*_B_ being the Boltzmann constant and *T*, the thermodynamic temperature), soft matter is typically characterized by a lower bonding energy, approximately on the order of the thermal energy, *k*_B_*T* (although certain specialized hydrogel systems, such as those containing double hydrophilic interpenetrating networks (double networks), can exhibit significantly higher energy scales). Soft matter systems, characterized by this small energy scale, are inherently dynamic, with thermal noise being sufficient to induce structural changes. This remarkable property enables soft matter systems to self-organize and self-heal and gives them a unique form of robustness through adaptability that is typically absent in traditional condensed matter systems. Such a small energy scale is typically related to the non-covalent interactions (like Van der Waals, ionic, hydrophobic, or hydrogen bonding) that hold soft matter together. It makes them, on the other hand, mechanically unstable because even small external forces can easily distort the system. However, recently, hydrogels with stimuli-responsive dynamic covalent bonds have emerged as a promising solution allowing hydrogels retain both mechanical stability and adaptability to external forces [2,3,4,5,6,7]. We will briefly review the field of stimuli-responsive dynamic covalent bonds in one of the upcoming sections, focusing on the types of crosslinks in hydrogels. Overall, this consideration underscores the fundamental reason why hydrogels are regarded as highly versatile and intriguing materials for a wide range of applications.

The initial synthesis and structural description of hydrogels were presented in 1960 by Wichterle and Lím, marking a foundational moment in hydrogel research [8]. Since then, hydrogel research has expanded significantly, with numerous scientists developing a broad spectrum of hydrogels featuring distinct properties. Within the various classes of hydrogels, one category includes those made from macromolecules derived from natural sources, such as nucleic acids [9]; cellulose, chitosan, and protein/peptide derivatives [10]; and polysaccharides [11]. Another category of hydrogels is formed by crosslinking macromolecules through covalent bonds. Examples include polyacrylates or polyacrylamides crosslinked with bis-acrylamides [12], boronate ester/diol linkers [13], or disulfides [14]. This crosslinking can also be achieved through the formation of supramolecular complexes [15] or metal–ligand complexes [16].

Micro-/nanogels, a macro- or nanoscale form of hydrogels, often referred to as hydrogel nanoparticles, combine the beneficial properties of both hydrogels and nanoparticles. Like larger hydrogels, nanogels exhibit high water content and responsiveness to environmental stimuli. However, their nanoscale dimensions offer additional advantages, including enhanced drug delivery, increased surface area, and improved penetration and interaction within biological systems. These gels can be synthesized through direct polymerization in both homogeneous and heterogeneous mixtures of different monomers, through self-assembly in solution, emulsion systems, or using template-based methods such as Particle Replication in Nonwetting Templates (PRINT) [17]. They can also be formed via polymerization processes that utilize larger polymeric structures as building blocks. Typically, the production methods involve grafting and/or crosslinking polymerization to create covalent bonds within the polymer backbone. For these processes, the substrate—whether a monomer or polymer—must contain at least one polymerizable functional group that reacts through radical initiation. Hydrogels and nanogels have so far demonstrated wide-ranging potential in on-demand applications [17,18]. In addition, nanogels provide an excellent platform for studying phase transitions in hydrogels, as the physical nature of the phase transitions (e.g., continuous or discontinuous) can vary depending on the form of the hydrogel (bulk or micro-/nanogels) [19].

A variety of physical and spectroscopic methods are employed to characterize hydrogels and nanogels. Key properties of these materials include their degree of swelling, surface porosity, and mechanical characteristics [18]. These features are influenced by the nature of the polymer backbone, the functional groups within the hydrogel or nanogel network, the density of crosslinking, and the capacity for water absorption. Various spectroscopic methods, including Nuclear Magnetic Resonance (NMR) spectroscopy, X-ray photoelectron spectroscopy (XPS), and Fourier transform infrared (FTIR) spectroscopy, are frequently utilized to analyze the structure and assess the crosslinking density of gelator matrices.

A distinct class of hydrogels, known as stimuli-responsive hydrogels, is particularly significant due to their ability to respond to various external stimuli [18]. Such hydrogels and nanogels can be regulated by a variety of physicochemical stimuli, including temperature [20,21,22], pH [23,24,25], light [26,27], electric [28,29,30] and magnetic [31,32,33] fields; redox chemical reactions [34,35,36,37]; ultrasound irradiation [38,39]; and various entities, including small molecules, proteins, enzymes, metal ions, and others [40,41,42,43,44]. These stimuli-responsive hydrogels have found extensive use in healthcare, serving roles in drug delivery systems for controlled or sustained release [45,46,47], sensing and biosensing [43,48,49,50], tissue and bone regeneration [51,52], bio-adhesives [53], hemostatic materials [54], and shape-memory applications [55,56]. They also play a key part in advanced applications such as actuation [57] and robotics [58].

One of the main challenges in designing hydrogels and nanogels lies in the precise quantification and adjustment of their functional groups [18]. Fine-tuning the side-chain functional groups is essential for creating responsive gels. Through integrating specific chemical groups, such as disulfide bonds, acid or amine groups, and azobenzene units, these hydrogels can be engineered to react to external stimuli.

This review summarizes recent advancements and innovations in the field of stimuli-responsive polymeric hydrogels, drawing on research from the past few years. Stimuli-responsive hydrogels are first categorized by the type of stimulus, with a focus on thermo-, pH-, light-, electro-, magneto-, and various entity-responsive systems. The fundamental properties of these hydrogels, such as swelling kinetics, mechanical strength, biocompatibility, and biodegradability, are then examined. Next, we explore various synthetic approaches for producing stimuli-sensitive hydrogels, highlighting strategies for fine-tuning their physicochemical properties and structural engineering. A comprehensive evaluation of their properties and effectiveness across diverse applications is provided. Finally, we address unresolved challenges in the field and offer perspectives for future research directions.

## 2. Classification of Stimuli-Responsive Hydrogels

Hydrogels can be classified based on various criteria. Here, we categorize stimuli-responsive hydrogels according to the stimuli they respond to, with a focus on their responsiveness to temperature, pH, light, electric and magnetic fields, and various entities.

### 2.1. Single-Stimulus Responsive Hydrogels

#### 2.1.1. Thermoresponsive Hydrogels

Thermoresponsive hydrogels (sometimes also referred to as thermogels) are a type of polymer that form gels by swelling them in water. Thermogelation in aqueous solutions arises from various mechanisms, some of which remain subjects of ongoing debate for specific polymers. A common characteristic of many thermoresponsive polymers is a decrease or increase in solubility caused by changes in the overall hydrophilicity of polymer chains in response to temperature fluctuations. In aqueous solutions, three primary types of interactions govern the behavior of thermoresponsive hydrogels [59]: (1) between polymer molecules, (2) between polymer and water molecules, and (3) between water molecules themselves. For polymers exhibiting a lower critical solution temperature (LCST), an increase in temperature results in a net unfavorable free energy for polymer–water interactions, thereby promoting polymer–polymer and water–water associations. This behavior can be understood thermodynamically through the relation Δ*G* = Δ*H* − *T*Δ*S*, where the free energy (Δ*G*) becomes negative due to a dominance of the entropy term (*T*Δ*S*) over the enthalpy term (Δ*H*) as temperature rises. The increase in entropy is primarily attributed to enhanced water–water associations, a phenomenon known as the hydrophobic effect. At the LCST, polymers undergo conformational changes, such as micelle packing or coil-to-helix transitions, which contribute to the formation of a gel network. These conformational shifts lead to a reversible physical linking of polymer chains, enabling the gel to revert to its original solubilized state upon the removal of the thermal stimulus. The LCST may depend on various parameters, but it typically decreases with increasing crosslinking density [60]. This behavior can be adjusted by varying the number of crosslinkers or the exposure dose during polymer photocrosslinking. Additionally, some thermoresponsive hydrogels exhibit volume phase transitions (e.g., shrinking or expanding as they transition between the swollen (liquid-like solution) and collapsed (solid-like gel) phases in response to temperature changes) and modifications in porosity or permeability, which influence solute diffusion. This dynamic and reversible process highlights the unique adaptability of thermogelling polymers and their potential applications in areas such as drug delivery, tissue engineering, and responsive materials. Conversely, the upper critical solution temperature (UCST) represents the temperature above which a polymer and solvent are fully miscible in all proportions, while below this threshold, phase separation occurs. UCST hydrogels exhibit a unique behavior, transitioning from an insoluble to a soluble state when the temperature exceeds this critical point. Complete miscibility is only achievable above the UCST [61]. The phase separation observed upon cooling an aqueous polymer solution is associated with the UCST and is primarily driven by enthalpy. In such systems, the interactions between polymer molecules and solvent molecules are weaker than the interactions among the polymer molecules themselves or among the solvent molecules [62]. Interestingly, some systems display both UCST and LCST behaviors [63]. These phase transitions are integral to the responsive properties of temperature-sensitive hydrogels, providing a means to control their solubility and swelling behavior based on temperature changes.

**Figure 1:**
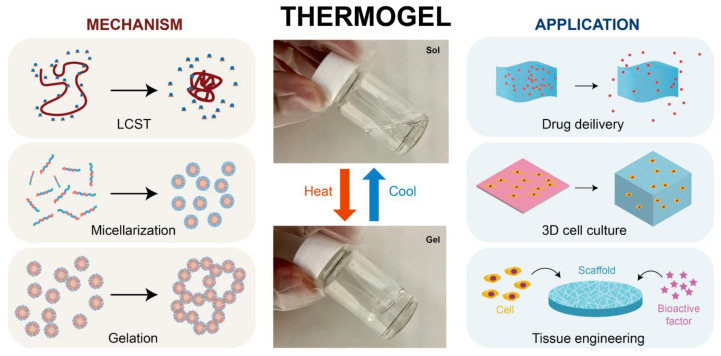
A schematic summary of the thermogelling behavior, illustrating the combined effects of multiscale thermoresponsive mechanisms, as well as the various applications of thermogels [65].

Typically, thermogels are composed of amphiphilic polymers containing both hydrophilic and hydrophobic segments, enabling them to undergo a sol–gel phase transition in response to temperature changes [64]. Unlike typical materials that melt at higher temperatures, some thermogels form gels when heated and revert to a liquid state as the temperature decreases within a specific range (see Figure 1) [65,66].

This phase transition occurs without the need for external triggers like enzymes, making the process environmentally friendly. Additionally, the absence of toxic crosslinkers enhances the biocompatibility of thermogels, making them suitable for use as injectable in situ hydrogels [67,68]. The application of thermogels extends across various fields, including drug delivery, where they are used to create controlled and sustained release systems due to their ability to undergo sol–gel transitions in response to temperature changes. In 3D cell culture, thermogels provide a supportive scaffold that mimics the extracellular matrix, facilitating cell growth and tissue formation. Additionally, in tissue engineering, these hydrogels play a crucial role by providing a biocompatible and injectable medium that supports cell proliferation and tissue regeneration, making them highly suitable for in situ tissue repair and regeneration. Their tunable properties and non-toxic nature further enhance their potential in these biomedical applications. Additionally, thermoresponsive hydrogels have been extensively investigated for the use in spectroscopy and biosensing [49].

#### 2.1.2. pH-Responsive Hydrogels

**Figure 2:**
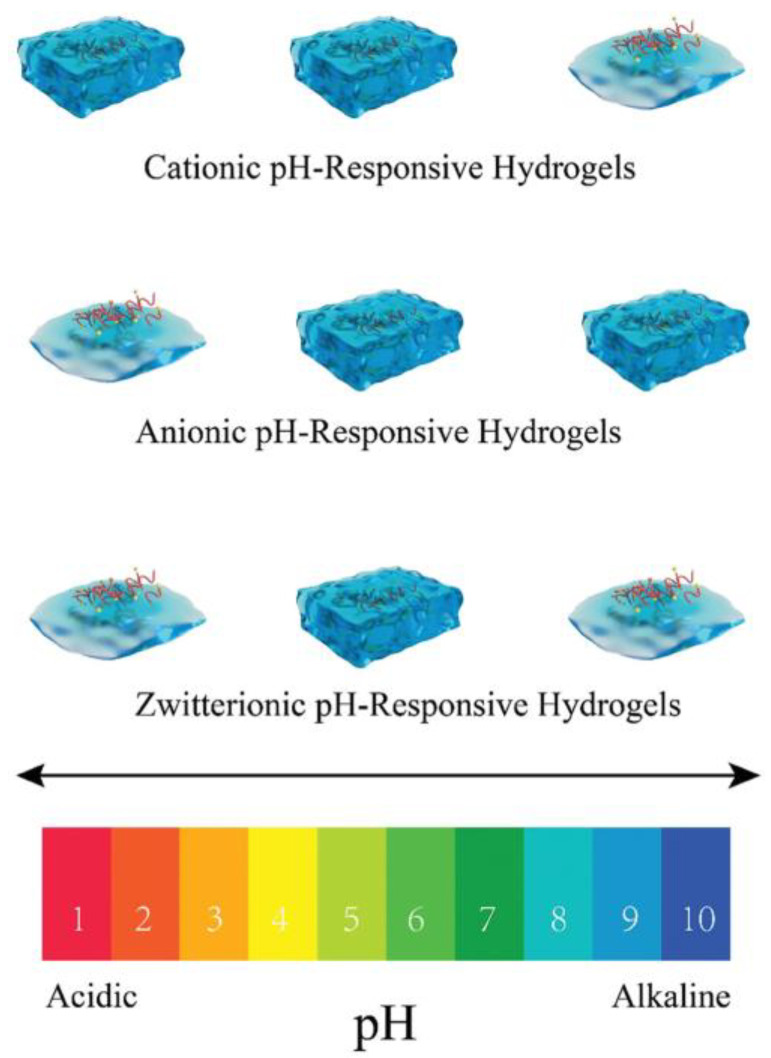
General working mechanism of pH responsive hydrogels [70].

pH-sensitive hydrogels are being intensively developed, with several factors influencing their swelling and deswelling behavior. These factors include ionic charge, the degree of ionization, the pH of the surrounding medium, monomer type, polymer concentration, and the hydrophilicity of the networks. In anionic hydrogels, weakly acidic functional groups (such as carboxylic −COO^−^, sulfonate −SO_3_^−^, and phosphate −PO_4_^2−^) ionize at higher pH levels, causing the network to swell. Conversely, at lower pH, cationic hydrogels swell due to the ionization of basic functional groups, such as amines (see Figure 2). The most important environmental applications of pH-responsive hydrogels include water treatment and purification, wastewater management, controlled release of agrochemicals, environmental sensing, and soil remediation [69]. The most important biomedical applications of pH-responsive hydrogels include drug delivery systems and cancer therapy, tissue engineering, wound healing, biosensing, and the controlled release of therapeutic agents [69].

#### 2.1.3. Photo-Responsive Hydrogels

**Figure 3:**
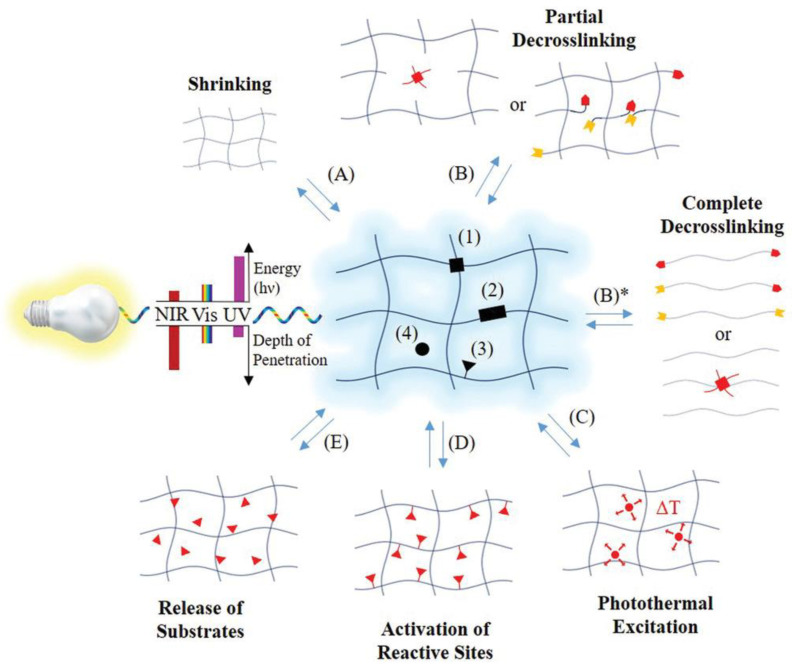
Molecular structure and responses of a photo-responsive hydrogel (center) [27]: The photo-responsive groups (black) can be located in various regions: (1) at crosslinking points, (2) within the polymer or supramolecular backbone, (3) alongside chains, or (4) dissolved within the hydrogel’s aqueous medium. Depending on the position and nature of the photo-responsive group, photo-induced responses may include shrinking (A) and partial de-crosslinking (B), typically associated with increased water absorption and, thus, greater hydrogel volume. Conversely, photo-triggered crosslinking tends to result in hydrogel contraction. Complete de-crosslinking leads to hydrogel breakdown (liquification) (B)*. Additional hydrogel responses include (C) photothermal activation (localized temperature increase), (D) the activation or deactivation of reactive sites, and (E) substrate release or capture.

Photo-responsive hydrogels generally comprise a polymer network integrated with a photoreactive component, often a photochromic chromophore that serves as the functional element. Initially, the optical signal is absorbed by the photochromic molecules [26,71,72]. The chromophores within the photoreceptor then transform the light signal into a chemical response through photoreactions such as isomerization, bond cleavage, or dimerization. This chemical signal is subsequently transmitted to the hydrogel’s functional component, regulating its properties. The response of the chromophores to light depends significantly on their molecular structure, and consequently, the required irradiation varies accordingly [27]. Photo-responsive hydrogels are utilized in creating dynamic cell microenvironments, enabling controlled drug release, designing adaptive surfaces, developing soft actuators, and producing healable soft materials [27]. The possible locations of the photo-active moieties within the hydrogel and the related possible photo-induced responses are shown in Figure 3.

#### 2.1.4. Electro-Responsive Hydrogels

Electrically sensitive, or electroactive, hydrogels and nanohydrogels are a distinctive class of materials capable of undergoing precise conformational changes—expanding or contracting—in response to an external electric field [18,28,29]. These hydrogels are often engineered for applications such as biomimetic artificial muscles. Their rapid and reproducible actuation makes them invaluable for drug delivery and biomedical engineering. The responsiveness of a hydrogel to an electric field can be tailored by adjusting the electrode placement relative to the hydrogel. Additionally, characteristics such as the hydrogel’s network-like structure, pore size, and the ionic strength of the surrounding solvent also play a role in determining the properties of electroactive hydrogels [18].

#### 2.1.5. Magneto-Responsive Hydrogels

Magneto-responsive hydrogels are advanced materials consisting of polymeric networks embedded with magnetic nanoparticles, such as iron oxide or other ferromagnetic materials [31,73]. These nanoparticles are uniformly dispersed within the hydrogel matrix, giving it the ability to respond dynamically to external magnetic fields. When subjected to a magnetic field, the nanoparticles align or generate localized heating [74,75], causing changes in the hydrogel’s mechanical, structural, or functional properties. These changes may include deformation, swelling, deswelling, or other dynamic behaviors.

Magneto-responsive hydrogels have a wide range of applications across various fields. In biomedical applications [31,76], they are used for targeted drug delivery, where the hydrogel releases therapeutic agents at specific sites under localized magnetic fields, improving precision and treatment efficacy. They also play a role in tissue engineering as scaffolds with adjustable mechanical properties that promote cell growth and differentiation [77]. Additionally, these hydrogels are used in hyperthermia therapy, where the magnetic nanoparticles generate heat under alternating magnetic fields, offering a non-invasive approach to cancer treatment [78,79,80]. In the field of soft robotics and actuators, magneto-responsive hydrogels are ideal for creating devices that can undergo reversible shape changes under magnetic stimuli, enabling the development of soft robotic systems, actuators, and artificial muscles [81,82,83,84]. They are also applied in sensors and diagnostic tools, where their responsiveness to magnetic fields is used to detect environmental changes, biological markers, or mechanical stress [85]. Environmental applications include their use in water purification and pollutant removal systems [73]. Their magnetic properties allow for the easy separation of the hydrogel after treatment. In addition, these hydrogels are used in the development of smart materials, such as responsive surfaces, tunable optical devices, and advanced coatings. Recently, the usage of magneto-responsive hydrogels has been extended to the investigation of the mechanotransduction in cells actuated by the magneto-responsive hydrogel structures [86,87]. To conclude, the versatility and responsiveness of magneto-responsive hydrogels make them a key material for innovative advancements in healthcare, robotics, and environmental technology.

#### 2.1.6. Entity-Responsive Hydrogels

Hydrogels, in addition to responding to various physicochemical stimuli, can also respond to specific entities such as metal ions [44], CRISPRs [42], small molecules and proteins [40], and glucose [41]. This responsiveness makes hydrogels highly valuable for detecting various entities and substances, offering significant potential for biosensing and environmental applications.

### 2.2. Multi-Stimuli Responsive Hydrogels

Multi-stimuli responsive hydrogels can undergo swelling changes when exposed to more than one of external factors such as pH, temperature, ionic strength, electric or magnetic fields, light, and chemical triggers [88]. They can undergo reversible or irreversible modifications in their physical properties and chemical structures. Multi-stimuli responsive hydrogels offer several advantages over single-stimulus responsive hydrogels due to their ability to react to multiple external triggers simultaneously or sequentially. These advantages include enhanced versatility, allowing them to function in more complex and dynamic environments. They provide precise and controlled responses, enabling fine-tuned changes such as swelling, shrinking, or drug release based on specific combinations of stimuli. Their higher specificity reduces unintended responses, improving reliability in applications like targeted drug delivery or biosensing. These hydrogels are also more stable and sensitive, maintaining functionality under a broader range of conditions. They have a wider application scope, including biomedical uses (e.g., targeted drug delivery [89] and tissue engineering), soft robotics, environmental remediation, and multifunctional sensors. In addition, multi-responsive hybrid particles, consisting of silica-coated magnetic particles encapsulated within a thermoresponsive hydrogel network on which gold rods are assembled, have been developed for advanced drug delivery applications [90]. Multi-stimuli hydrogels enable the development of smart systems that mimic natural behaviors by responding to combinations of inputs, such as temperature, pH, or mechanical stress. They also exhibit synergistic effects, achieving behaviors that are not possible with single-stimulus hydrogels, such as complex shape transformations, directional motion, or synergistic chemo-magnetic hyperthermia tumor therapy [91]. Dual-responsive hydrogels offering photo-reversible luminescence switch and thermos-reversible anisotropic deformation have also been demonstrated for the multidimensional intelligent anticounterfeiting applications [92]. Overall, the adaptability and multifunctionality of the multi-stimuli-responsive hydrogels make them highly valuable in advanced material applications.

## 3. Properties of the Hydrogels and Approaches for Their Design, Synthesis, and Structure Engineering

Having classified hydrogels based on their responsiveness to various stimuli, it is valuable to first delve into the fundamentals of their key properties, the factors that influence these properties, and the strategies for their design, synthesis, and structural engineering. This foundational understanding provides essential context for evaluating their applications.

### 3.1. Swelling Kinetics

**Figure 4:**
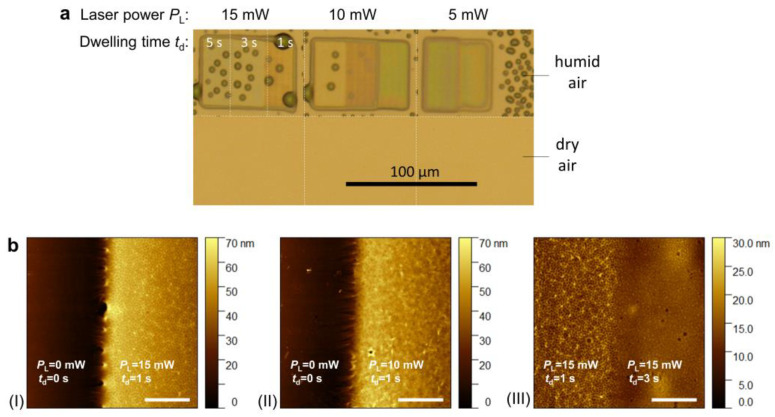
(**a**) Bright-field optical microscopy images of structures made from poly(NIPAAm_95_-*co*-BPQAAm_5_) are shown for the specified combinations of *P*_L_ and *t*_d_, captured in dry air (bottom row) and in humid air to induce hydrogel formation (top row). (**b**) AFM images of the same poly(NIPAAm_95_-*co*-BPQAAm_5_) structures are presented at the interface between the bare gold substrate and the polymer layer, prepared under the following conditions: (I) *P*_L_ of 15 mW and *t*_d_ of 1 s; (II) *P*_L_ of 10 mW and *t*_d_ of 1 s; and (III) between two polymer network regions fabricated with *P*_L_ of 15 mW, *t*_d_ of 1 s, and *P*_L_ of 15 mW, *t*_d_ of 3 s. The scale bar represents 5 µm [93].

The swelling behavior of hydrogels in various environments, such as water or biofluid, is a defining characteristic that enables their use in diverse fields. As outlined in the previous sections, stimuli-responsive hydrogels respond to changes in biological and environmental conditions, including variations in temperature, pH, ionic composition, solvent type, electric field, and light exposure. Swelling kinetics and equilibrium are influenced by factors such as the crosslinking ratio, polymer chemical composition, ionic environment, and synthesis conditions. The degree of swelling *Q*, which quantifies the swelling of a hydrogel, is defined as $Q=\left(V_{s}-V_{d}\right)/V_{s}$, where *V*_s_ and *V*_d_ are the swollen and dry volumes, respectively. Figure 4 presents an example of a poly(*N*-isopropyl acrylamide) [pNIPAAm]-based hydrogel under different environmental conditions, specifically in dry air and humid air [93]. Additionally, atomic force microscopy (AFM) images illustrate its wrinkle-like structure observed under certain photocrosslinking parameters. As shown in Figure 4a, the created polymer networks are, in some cases, challenging to observe in their dry state (prior to swelling due to the contact with water molecules or other hydrating agents).

Crosslinking plays a crucial role, as highly crosslinked hydrogels exhibit lower swelling ratios due to restricted network expansion, while loosely crosslinked structures swell more extensively. The chemical structure of the hydrogel also significantly impacts its swelling properties; polymers with more hydrophilic groups swell to a greater extent compared to those with hydrophobic groups. Temperature and pH further affect swelling. pH-sensitive hydrogels swell as hydrophilic groups ionize with changes in pH, generating electrostatic repulsion between like charges and disrupting secondary bonds within the polymer network.

**Figure 5:**
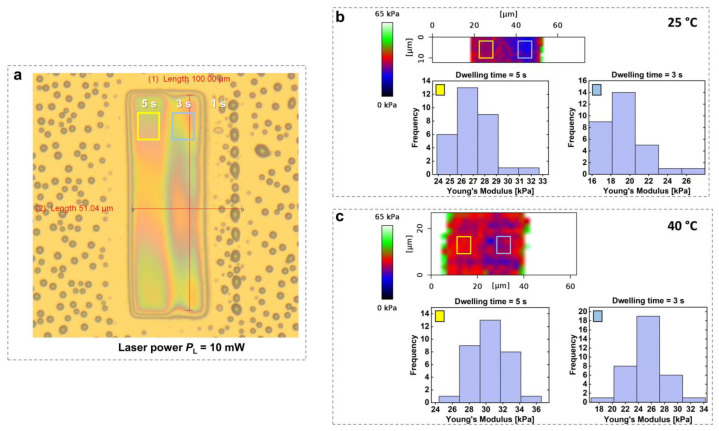
Temperature-controlled AFM measurements of Young’s moduli performed for a poly(NIPAAm_94_-co-MAA_5_-co-BPQAAm_1_) hydrogel pad prepared using a laser power *P*_L_ of 10 mW and dwelling times *t*_d_ of 1, 3, and 5 s [93]. (**a**) A bright-field microscopy image shows the formed hydrogels and water condensation patterns after exposure to humid air. (**b**,**c**) Histograms display the measured Young’s moduli at 25 °C and 40 °C, respectively. The regions marked with blue and yellow rectangles in the microscopy image indicate the areas where the modulus distributions were analyzed.

Temperature-sensitive hydrogels undergo a volume phase transition (between a liquid-like solution phase and solid-like gel phase) at the volume phase transition temperature (VPTT), resulting in either the swelling or collapsing of the hydrogel network depending on the environmental conditions. It is worth noting that alternative terms for VPTT, such as “gel collapse temperature” or “gel shrinkage temperature”, are also commonly used in the literature [94]. Figure 5 shows an example of a pNIPAAm-based temperature-responsive hydrogel in aqueous solution at different temperatures [93]. Figure 5b,c indicate that for hydrogels prepared with *t*_d_ = 3 s and *t*_d_ = 5 s at an irradiation power *P*_L_ of 10 mW, Young’s modulus increased from 19 to 26 kPa and from 26 to 30 kPa, respectively, as the temperature rose from 25 °C to 40 °C. This increase reflects the collapse of the hydrogel microstructures at temperatures exceeding the LCST of pNIPAAm, resulting in a denser and stiffer polymer network.

Swelling occurs in three stages: (1) water diffuses into the hydrogel network, (2) the polymer chains loosen, and (3) the hydrogel network expands. In its dehydrated state, the hydrogel exists in a “glassy” state, while in its swollen form, it transitions to a “rubbery” state. The free spaces within the polymer network allow solvent molecules to infiltrate when a dry or glassy hydrogel comes into contact with an aqueous medium. Once sufficient water has permeated the matrix, the transition from a glassy to rubbery state, termed swelling, is complete. This diffusion process governs both the uptake and release of water within the hydrogel matrix.

Swelling kinetics of hydrogels describes the rate and mechanism by which they absorb solvent and reach an equilibrium swollen state. These kinetics can be categorized as first-order swelling kinetics and second-order swelling kinetics, depending on the relationship between the rate of swelling and the degree of swelling achieved. In first-order kinetics, the rate of swelling is proportional to the difference between the equilibrium swelling degree *Q_eq_* and the current swelling degree *Q*(*t*) at time *t*:

(1) $$\frac{dQ(t)}{dt}=k_{1}\left(Q_{eq}-Q(t)\right),$$

where *k*_1_ is the first-order swelling rate constant. In assuming that the swelling degree is zero at *t* = 0, Equation (1) can be integrated to yield the following solution:

(2) $$Q(t)=Q_{eq}\left(1-e^{-k_{1}t}\right).$$

Hydrogels exhibiting first-order swelling kinetics show rapid initial swelling due to the fast diffusion of water into the polymer matrix, followed by a slower, exponential approach to equilibrium as the network gradually expands and the swelling rate decreases. Typically, this is governed by diffusion-limited processes, where solvent penetrates the hydrogel and gradually expands the polymer network. Diffusion models describe how solvent molecules diffuse into the hydrogel network, causing it to swell. The process is typically driven by a gradient in chemical potential between the hydrogel and the surrounding solvent. Three key diffusion mechanisms are often considered: Fickian diffusion, where the rate of swelling is determined by the diffusion of solvent into the polymer network; Case II diffusion, which involves solvent transport coupled with polymer chain relaxation; and anomalous diffusion, where both mechanisms contribute simultaneously. For a more detailed explanation of the diffusion models, we recommend referring to the comprehensive work by Berens and Hopfenberg [95]. These models provide valuable insights into the dynamics of hydrogel swelling and are critical for designing materials with specific swelling behaviors. First-order swelling kinetics is common in hydrogels where the polymer chains are highly crosslinked (=rigid networks), and swelling is limited by the time it takes for solvent to diffuse. Hydrogels with first-order swelling kinetics can be beneficial in applications such as special drug delivery systems, where rapid solvent absorption is critical for initial drug release, or systems designed to respond quickly to environmental changes (e.g., thermo- or pH-responsive hydrogels).

In second-order kinetics, the swelling rate depends on the square of the difference between the equilibrium swelling degree *Q_eq_* and the current swelling degree *Q*(*t*):

(3) $$\frac{dQ(t)}{dt}=k_{2}{\left(Q_{eq}-Q(t)\right)}^{2},$$

where *k*_2_ is the second-order swelling rate constant. The solution to Equation (3) is

(4) $$\frac{1}{Q_{eq}-Q(t)}=\frac{1}{Q_{eq}}+k_{2}t.$$

According to Equation (4), second-order swelling is characterized by a slower initial swelling compared to first-order kinetics, with the swelling rate gradually accelerating before approaching equilibrium. Typically, this reflects a polymer relaxation-controlled process, where the swelling is limited by the ability of the polymer network to rearrange and accommodate the solvent. Second-order swelling kinetics is common in lightly crosslinked hydrogels or systems where polymer mobility is the limiting factor and often seen in hydrogels with flexible networks or weak physical crosslinks. Hydrogels with second-order swelling kinetics can be beneficial for tissue engineering or long-term drug release, where a controlled, slower swelling rate is desirable. In addition, they can find applications in soft gels that mimic biological systems with gradual, time-dependent changes.

### 3.2. Mechanical Properties

The properties of a specific hydrogel play a crucial role in determining its suitability for a given application. However, these properties are highly influenced by the surrounding environmental conditions. Therefore, it is essential to measure the hydrogel properties under conditions that closely replicate their *in situ* environment. In some cases, testing a hydrogel under the exact conditions of its intended application is not feasible. In such instances, it becomes particularly important to employ theoretical models to extrapolate its properties to those conditions. The mechanical behavior of hydrogels is best understood using the theories of rubber elasticity and viscoelasticity [96]. These theories are based on the time-independent and time-dependent recovery of chain orientation and structure, respectively.

Rubbers are materials that exhibit nearly instantaneous and fully reversible deformation in response to stress [97]. While glasses can be reversibly stretched by only up to 1%, rubbers typically demonstrate reversible behavior at elongations of up to ten times. Normal rubbers consist of lightly crosslinked networks with a significant free volume, allowing for the rapid rearrangement of polymer segments under external stress. In their swollen state, most hydrogels meet these criteria for rubber-like behavior. When a hydrogel operates within this rubber-like region, its mechanical properties are primarily determined by the architecture of its polymer network. The general characteristics of rubber elastic behavior include high extensibility under low mechanical stress, complete recovery upon the removal of deformation, and the fact that both extensibility and recovery are predominantly driven by entropic changes rather than enthalpic changes. Based on classical or statistical thermodynamics, the retraction force of the rubber-like hydrogels in response to a tensile force can be estimated [96].

At sufficiently low temperatures, these gels can lose their rubber-elastic properties and instead exhibit viscoelastic behavior. The theory of viscoelasticity examines the interplay between elasticity, flow, and molecular motion in polymeric materials. While all materials exhibit some degree of elasticity and flow, the large size of polymer molecules often results in a pronounced viscoelastic response. The magnitude of this response strongly depends on the nature of the imposed mechanical motion. For instance, the viscoelastic response reaches its maximum when the time scale of the mechanical motion matches the time scale of the polymer’s molecular motion. Hydrogels, in general, are not purely elastic materials but exhibit viscoelastic behavior. Therefore, the time dependence of the applied stress or strain is as critical as its magnitude when predicting the material’s mechanical response. An applied mechanical stress (or strain) induces a time-dependent response in strain (or stress) as the segments of the polymer chains adjust their positions. This movement generates an internal response, leading to a time-dependent recovery when the initial stress or strain is removed. If the recovery is complete over a sufficiently long time, the behavior is classified as elastic. If the recovery is incomplete, the behavior is termed viscoelastic. In general, a polymer may exhibit multiple viscoelastic processes, each associated with a specific type of molecular motion within the material [96]. These mechanical measurements, collectively referred to as rheology, are critically important for determining the properties of stimuli-responsive hydrogels under various conditions.

The six most common techniques for testing the mechanical properties of hydrogels include tension and compression (which can be performed in either unconfined or confined setups), local indentation using a probe, and frequency-based methods such as shear rheometry and dynamic mechanical analysis (DMA) [98]. Additionally, mechanical properties of the hydrogels can be measured by non-invasive dynamic light scattering [99]. Alternatively, recent studies by Bailey et al. [100] and Mahmodi et al. [101] have demonstrated that non-contact Brillouin microscopy/spectroscopy is a suitable technique for inferring the mechanical properties of hydrogels. In the case of rubber elastic behavior, tensile testing is most commonly used. At the same time, DMA is one of the most developed techniques available today for obtaining quantitative information on the viscoelastic and rheological properties of a material. This is accomplished by measuring the mechanical response of a sample when subjected to periodic stress or strain. The generalized notation for sinusoidal tests is the complex dynamic modulus G* [96]:

(5) $$G^{*}=G\prime +iG\prime \prime =\frac{\sigma^{*}}{\gamma^{*}},$$

where *G*′ represents the real part of the modulus, also known as the elastic or storage modulus, and *G*′′ represents the imaginary part, referred to as the viscous or loss modulus. These definitions are applied in the most common shear mode testing, where *G* denotes the shear modulus, *σ* corresponds to the shear stress, and *γ* refers to the shear strain. Measured values of *G*′ and *G*′′ give us the ability to calculate different properties of the hydrogels, e.g., the number-average molecular weight between crosslinks $\bar{M_{c}}$ as $\bar{M_{c}}=\rho RT/G\prime$, where *ρ* is the density, *R* is the ideal gas constant, and *T* is the temperature.

For stimuli-responsive hydrogels, rheological measurements provide valuable information about the impact of external stimuli on network stability and dynamics. For instance, temperature-responsive hydrogels such as poly(*N*-isopropylacrylamide) exhibit significant changes in *G*′ and *G*′′ as they undergo a phase transition [102]. Similarly, light-responsive hydrogels often show tunable viscoelastic properties depending on the light intensity or wavelength. These dynamic modulations are often reversible, making rheology a key tool for characterizing the mechanical adaptability of such materials.

**Figure 6:**
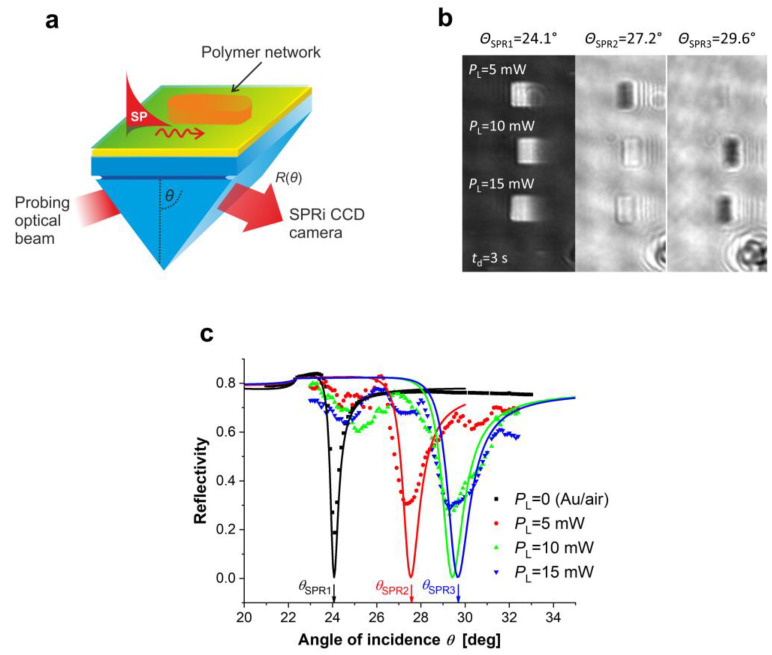
(**a**) Schematic representation of the SPRi setup used for probing the pNIPAAm-based microstructures in air. (**b**) SPRi images of the microstructures. (**c**) Corresponding angular reflectivity spectra in air for poly(NIPAAm_95_-*co*-BPQAAm_5_) structures prepared with a constant dwelling time *t*_d_ = 3 s and varying laser power *P*_L_ = 5, 10, or 15 mW. The solid curves in (**c**) are fits calculated using a Fresnel-based reflectivity model [93].

Mechanical properties of the hydrogels can be controlled with the crosslinking density, degree of swelling, polymerization/crosslinking conditions, and co-monomer composition, to name a few. For example, in the case of the multiphoton photocrosslinking of a pNIPAAm-based thermoresponsive hydrogel, increasing the crosslinking femtosecond laser power leads to a more optically dense and rigid polymer networks, which was revealed by the surface plasmon resonance imaging (SPRi) technique (see Figure 6) [93]. Khan et al. showed [103] that the mechanical properties of poly(acrylic acid) hydrogels can be alternated by the charge density. More detailed and advanced descriptions of the discussed measurement techniques, theoretical models, and the methods for the mechanical properties control can be found in [96,98,104] and the references therein.

### 3.3. Hydrogel Design, Synthesis, and Structure Engineering

Before discussing the biocompatibility and biodegradability of hydrogels—key parameters in the context of their applications—it is valuable to first examine the approaches used for polymer synthesis, network formation, and subsequent structural engineering. These factors play a critical role in shaping the biocompatibility and biodegradability of the resultant hydrogels.

Crosslinks in a hydrogel refer to the forces or bonds that connect polymer chains, forming a three-dimensional network. These crosslinks determine the structure and properties of the hydrogel, including its mechanical strength, elasticity, swelling behavior, and biodegradability. Crosslinking forces can be classified into two broad categories: chemical crosslinks and physical crosslinks.

#### 3.3.1. Physical Crosslinking

**Figure 7:**
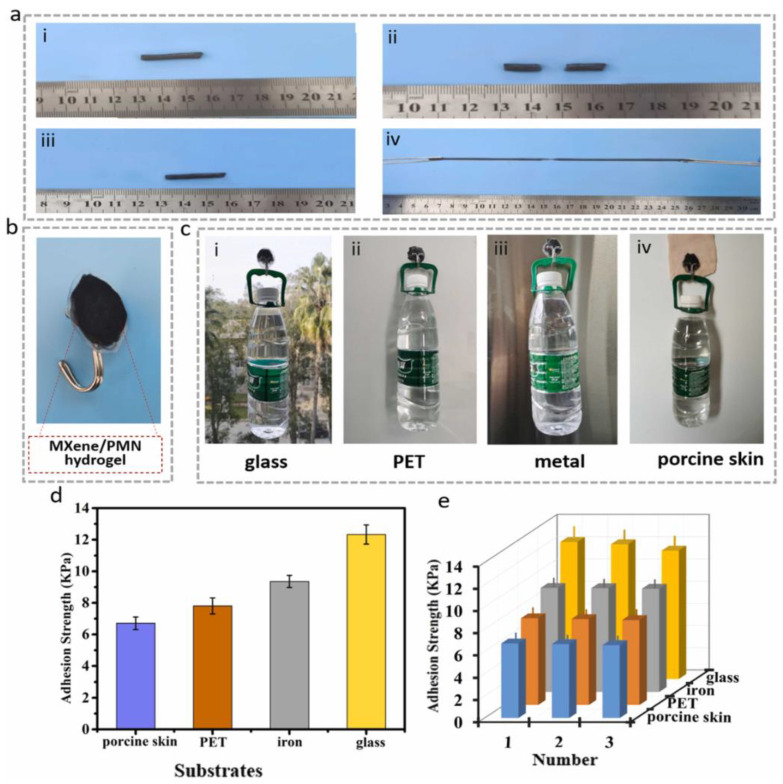
Self-healing and self-adhesion performance of the MXene/PMN hydrogel [113]. (**a**) Photographs illustrating the self-healing process of the MXene/PMN hydrogel: (i) original state, (ii) after being cut, (iii) during the self-healing process, and (iv) stretched after self-healing. (**b**) A hook coated with MXene/PMN hydrogel. (**c**) The MXene/PMN hydrogel hook adhered to various surfaces: (i) glass, (ii) PET, (iii) metal, and (iv) porcine skin. (**d**) Self-adhesive strength of the MXene/PMN hydrogel on different substrates. (**e**) Repeatable self-adhesive behavior of the MXene/PMN hydrogel on various substrates. Reprinted from [113] with permission from Elsevier.

Physical crosslinking refers to the formation of hydrogel networks through non-covalent interactions such as hydrogen bonding, ionic interactions, hydrophobic associations, van der Waals forces, and physical entanglements [105,106]. Unlike chemical crosslinking, physical crosslinking avoids the use of covalent bonds and toxic crosslinkers, leading to biocompatible systems that are largely reversible. Internal non-covalent interactions can be stimulated/induced by external factors such as temperature [107] and pH [108]. Other external triggers include ionic strength, mechanical stress, and the presence of specific chemicals or biomolecules [109], depending on the nature of the hydrogel and its functional groups. These stimuli influence the physical interactions (e.g., hydrogen bonding, ionic interactions, hydrophobic associations, or host–guest chemistry) that govern the structure and properties of physically crosslinked hydrogels. An intriguing feature of physically crosslinked hydrogels is that the physical interactions governing the polymer network can be modulated or altered by external stimuli. For instance, Zhang et al. demonstrated a pH-induced mechanical transition in polyion complex hydrogels, where the network shifted from ionic associations to hydrophobic associations [110]. Typically, physical hydrogels, which lack covalent bonds, are considered to have low mechanical strength and stability. However, polyampholytes—charged polymers containing both positively and negatively charged groups [111]—can serve as a basis for overcoming this limitation. Sun et al. showed that physical hydrogels composed of polyampholytes exhibit high toughness and viscoelasticity [112]. In this system, multiple ionic bonds are formed due to the randomly arranged charge distribution. Among these, the strong ionic bonds act as permanent crosslinks, maintaining the overall structural integrity of the hydrogel, while the weaker ionic bonds function as sacrificial crosslinks. These sacrificial bonds contribute to the hydrogel’s excellent self-healing properties, fracture resistance, and enhanced toughness. Polyampholyte hydrogels can be combined with other high-performance materials, such as MXene, to further enhance their properties and expand their range of applications (Figure 7) [113]. The preparation of hydrogels utilizing a combination of physical crosslinks of different nature has been demonstrated by several research groups [114,115,116]. This opens the door to a more versatile use of hydrogels, allowing them to adapt to changes in their environment. Another important subclass of physical hydrogels is stimuli-responsive supramolecular hydrogels [117,118,119,120,121,122]. These hydrogels are formed from low-molecular-weight gelators or polymeric building blocks that self-assemble into three-dimensional networks through non-covalent interactions. Common building blocks include peptides, cyclodextrins, dendrimers, or other organic molecules capable of forming hydrogen bonds, π-π stacking, or host–guest interactions. These non-covalent interactions endow the hydrogels with unique stimuli-responsiveness, enabling them to dynamically respond to external triggers such as pH, temperature, light, or specific chemical signals. Their reversible and tunable properties make them highly versatile and attractive for a wide range of applications.

#### 3.3.2. Chemical Crosslinking

Chemically crosslinked hydrogels are polymeric networks formed through covalent bonds between polymer chains. These hydrogels exhibit enhanced mechanical strength, stability, and structural integrity, making them ideal for some applications in biomedical, environmental, and industrial fields. Mechanisms of chemical crosslink formation include free radical polymerization, photocrosslinking, click chemistry, enzymatic crosslinking, and chemical reagents. These and some other mechanisms have been recently reviewed in detail in, e.g., Ref. [82].

**Figure 8:**
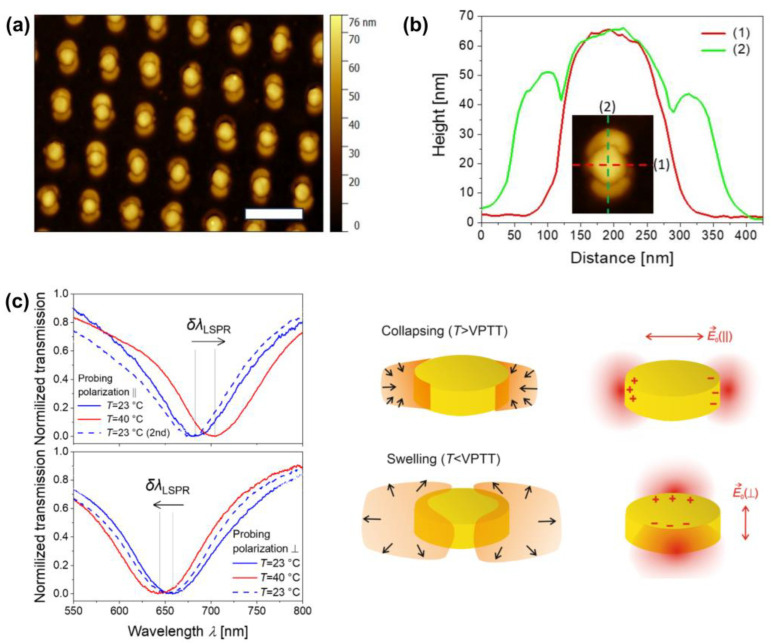
Plasmonically enhanced multiphoton polymer crosslinking (MPC) [126]: (**a**) AFM height topography image of the crosslinked polymer at the plasmonic hotspots with (**b**) respective cross sections for individual gold nanoparticle—polymer structure; (**c**) normalized transmission spectra of the plasmonically enhanced MPC-written poly(NIPAAm_94_-*co*-MAA_5_-*co*-BPQAAm_1_) sample and schematics of the hydrogel swelling and collapsing at the plasmonic hotspots.

In addition, the multiphoton polymerization of monomer solutions has been demonstrated as a suitable method for fabricating interpenetrating polymer networks with customized microstructures, thermal properties, and micromechanical characteristics [123]. However, here, we would like to briefly highlight an alternative mechanism for polymer network formation that is capable of producing hydrogels upon exposure to water. This method, often overlooked in the literature, holds great potential for applications due to its versatility. Unlike the traditional approach, it is based on the multiphoton crosslinking of pre-synthesized polymers rather than monomer solutions. In the original paper [124], two-photon absorption was shown to induce C,H-insertion reactions, resulting in strictly localized crosslinking within the polymer. With this approach, in scanning a near-infrared (NIR) beam, the protein-repellent and protein-adsorbing polymers have been fabricated, enabling the resulting 3D structure to exhibit spatially controlled protein adsorption. Later, Morozov et al. extended the approach to enable the microstructuring of pNIPAAm-based thermoresponsive biofunctional hydrogels [93]. In the context of biofunctional structures, using an NIR beam for hydrogel crosslinking offers the added advantage of minimizing the risk of damaging delicate functional groups typically associated with UV light exposure. Additionally, this approach enables the fabrication of structures from a compact dry polymer layer, eliminating the risk of residual monomers or photoinitiators leaching out when the hydrogel swells. It also offers the advantage of easily incorporating multiple functionalities during the polymer pre-synthesis step, such as the ability to post-modify the hydrogel structure with biomolecules, as demonstrated in [93]. Moreover, the solid, uncrosslinked polymer layer serves as a support material during the writing of complex 3D structures, a challenge often faced in the photopolymerization of liquid monomers, which cannot support overhanging geometries. This approach becomes even more attractive when considering that the optical system for direct laser writing can, in this case, be implemented more simply, requiring a minimal number of optical components [125]. Recently, this concept of the multiphoton crosslinking of pre-synthesized polymers has been extended to plasmonic nanostructures, where plasmonically enhanced multiphoton polymer crosslinking enables the localized modification of plasmonic hotspots with thermoresponsive biofunctional hydrogels at nanometer-scale resolution. Figure 8 illustrates a hybrid metallic–hydrogel material created using this method, along with its temperature-dependent reversible behavior, as demonstrated through polarization-resolved LSPR spectroscopy [126].

While chemical gels are commonly regarded as rigid and less adaptable to stimuli compared to physical gels, it is worth highlighting a general approach known as “structurally dynamic hydrogels” [127]. This approach includes hydrogels based on stimuli-responsive dynamic covalent bonds [3,5,6,7]. These hydrogels achieve a balance between macroscopic stability and microscopic dynamics, thereby overcoming the limitations typically associated with covalent bonds.

Finally, when designing a hydrogel, one can consider incorporating chemical crosslinks, physical crosslinks, or a combination of both (hybrid crosslinks) [128,129].

### 3.4. Biocompatibility and Biodegradability

Hydrogels, characterized by their high water content, are generally considered safe for living organisms and biological systems. This safety, however, is not an inherent property but is highly dependent on multiple factors. These include the type of polymer network, its constituent components, the nature of the crosslinks (physical, chemical, or hybrid), their density, and other structural parameters that influence the hydrogel’s behavior. The physical and chemical properties of the polymer itself—such as melting point, glass transition temperature, storage modulus, and crystallinity—are critical in determining not only the hydrogel’s mechanical and thermal behavior but also its biodegradability [130]. For instance, polymers with lower crystallinity and higher amorphous regions often exhibit enhanced biodegradability due to their susceptibility to enzymatic and hydrolytic degradation.

As discussed in the previous subsections, physical hydrogels are often regarded as more biocompatible and biodegradable compared to their chemically crosslinked counterparts. This distinction arises primarily from the absence of strong covalent bonds in physical hydrogels, which allows for dynamic interactions and reversible assembly under physiological conditions. Consequently, physical hydrogels are better suited for applications requiring high biocompatibility, such as drug delivery, tissue engineering, and wound healing. By contrast, chemically crosslinked hydrogels, while offering greater mechanical stability, may pose challenges in terms of biocompatibility and degradability due to the persistence of covalent bonds that resist natural or artificial degradation processes.

New biocompatible and biodegradable hydrogel designs are often inspired by nature and naturally occurring hydrogels [131,132,133,134]. These natural systems, such as extracellular matrices, mucilages, and other biohydrogels found in plants and animals, provide invaluable blueprints for creating advanced hydrogel materials. By mimicking their structural, mechanical, and functional properties, researchers aim to replicate the dynamic adaptability, selective permeability, and self-healing capabilities of natural hydrogels. This biomimetic approach not only enhances the biocompatibility and degradability of synthetic hydrogels but also broadens their potential applications in areas such as tissue engineering, drug delivery, and environmental remediation.

In the case of the widely used pNIPAAm thermoresponsive hydrogel [130], the concentration of NIPAAm molecules and the number of crosslinks significantly influence its biocompatibility and biodegradability. Consequently, the synthesis of pNIPAAm-based thermoresponsive hydrogels incorporating renewable natural resources can be considered a promising approach to enhancing these properties. Koca et al. showed [135] that incorporating bacterial cellulose (BC) and castor oil (CO), which are known by their biocompatible and biodegradable characteristics [136,137,138,139], despite reducing the concentration of NIPAAm molecules in the resultant hydrogel, also improve its swelling/deswelling properties. In another study, with a simple and robust mixing without need for complicated derivatization, a pNIPAAm–cellulose nanocrystal hydrogel as thermoresponsive and biocompatible material was produced [140]. A good overview of other temperature-responsive hydrogels with respect to their biocompatibility/biodegradability is given in [141].

To summarize, this inherent variability underscores the importance of tailoring hydrogel properties to specific applications, ensuring an optimal balance between structural integrity, biodegradability, and compatibility with biological systems [142]. Such adaptability positions hydrogels as versatile materials for diverse biomedical and environmental applications.

## 4. Applications of Stimuli-Responsive Hydrogels

In our discussion of the properties of stimuli-responsive hydrogels, we already highlighted several potential areas of application. Here, we aim to provide a more detailed exploration of their use across both well-established and emerging fields.

### 4.1. Biomedical Applications

Biomedical applications encompass a wide range of fields, including biosensing, drug delivery, tissue engineering, and wound healing. In recent decades, hydrogels and nano/microhydrogels have been extensively explored for these applications [43,143,144,145,146,147,148]. In all these applications, in addition to the commonly shared features of all hydrogels, such as high surface area and substantial water intake, stimuli-responsive hydrogels provide additional advantages owing to their responsiveness. These include more controlled drug release, enhanced sensitivity, improved adaptability to environmental changes, and the ability to perform specific functions in response to external stimuli such as temperature, pH, light, or biochemical signals. Since effective medical treatment relies on the early screening and detection of pathologies, we will begin by discussing biosensing, which focuses on the recognition and detection of biologically significant substances, before exploring other biomedical applications.

#### 4.1.1. Biosensing

In biosensing, the responsive hydrogels can first serve as a binding matrix for incorporating the analyte-capturing biomolecules with inherent molecular recognition properties (e.g., proteins, peptides, enzymes) into the polymer network. Then, the hydrogel matrix can be changing by alternating an external stimulus (such as temperature or pH) leading to the change in the detected signal. In a surface plasmon-enhanced fluorescence spectroscopy study [49], a pNIPAAm-based hydrogel matrix was modified with mouse immunoglobulin G (mIgG). Upon capturing fluorophore-labeled goat anti-mouse IgG (a-mIgG), the hydrogel matrix could be shrunk by raising the temperature above its volume phase transition temperature (VPTT). This process results in significant refractive index changes correlated with the amount of captured analyte, which can be observed as a shift in the SPR signal. Furthermore, the shrinking hydrogel brings the fluorophore closer to the gold SPR sensor surface, enhancing the fluorescence signal through plasmonic effects. Later, Hageneder et al. [149] further improved this configuration by organizing the responsive hydrogels into sensing spots for detecting human IgG antibodies against the Epstein–Barr virus in plasma or isolated IgG, using peptide ligands mapping the EBV nuclear antigen. Low-molecular-weight peptide ligands were coupled via click chemistry, preserving the thermoresponsive properties, unlike higher-molecular-weight ligands such as antibodies. This approach underscores the potential for improved fluorescence biosensing. As previously mentioned, Morozov et al. demonstrated the ability to perform plasmonically enhanced the crosslinking of thermoresponsive biofunctional hydrogels at plasmonic hotspots [126]. In leveraging the thermoresponsiveness of the hydrogels, this approach enables the creation of effectively extended hotspots, offering significant potential in advancing bioanalytical applications. Numerous approaches utilizing stimuli-responsive hydrogels have been proposed for cancer detection [150] and food safety [151]. Bhat et al. developed a pH-responsive poly(2-hydroxyethyl methacrylate) [poly(HEMA)] hydrogel as an impedimetric sensor for monitoring tissue acidosis caused by hemorrhagic trauma [152]. Four hydrogel formulations were tested: cationogenic primary amine (AEMA), tertiary amine (DMAEMA), and a combined AEMA–DMAEMA system. Electrochemical impedance spectroscopy revealed that the AEMA hydrogel, particularly at 1 mol%, exhibited the highest sensitivity in the pathophysiological pH range (7.35–7.45). The AEMA hydrogel was optimized using the Taguchi Design of Experiments and Response Surface Methodology, confirming 1 mol% AEMA as the most robust and sensitive formulation. This hydrogel was integrated into a microlithographically fabricated interdigitated microsensor electrode, with sensitivity assessed using R(QR)(QR) modeling. Gravimetric analysis and differential scanning calorimetry showed a strong anticorrelation between nonfreezable bound water and pH sensitivity, identifying bound water as the key factor influencing hydrogel responsiveness. Endo et al. proposed a stimuli-responsive hydrogel–silver nanoparticle composite for developing a localized surface plasmon resonance (LSPR)-based optical enzyme biosensor [153]. The biosensor, constructed by immobilizing glucose oxidase (GOx) into the hydrogel, detects glucose by changes in LSPR absorbance. When glucose interacts with the biosensor, the enzymatic reaction produces hydrogen peroxide, causing the degradation of clustered silver nanoparticles and altering interparticle distances. This results in a significant LSPR absorbance change proportional to the glucose concentration, with a detection limit of 10 pM. This cost-effective and highly sensitive biosensor shows promise for simplified medical diagnostic applications. Tokarev et al. combined three distinct phenomena—biocatalysis, localized surface plasmon resonance (LSPR) in noble metal nanoparticles, and the swelling–shrinking transition of a stimuli-responsive hydrogel—to create a novel sensing platform [154]. This platform was designed for two key applications: biomolecule analysis (biosensors) and monitoring the local properties of biomaterials. The integration of these mechanisms enables efficient biochemical-to-optical signal transduction with high specificity, while minimizing the influence of nonspecific interactions. Another important type of biosensor relies on analyte- or target-responsive hydrogels, which, in addition to typical swelling or syneresis responses, exhibit unique behaviors such as gel assembly or disassembly upon interaction with the target analyte [43]. Yang et al. are among the pioneers in this field, demonstrating that the competitive binding of a target molecule to an aptamer decreases the crosslinking density of the hydrogel, leading to its dissolution [40]. This strategy was successfully adapted for therapeutic applications using two types of targets: small molecules and proteins. The results showcased the potential of this molecular engineering approach to create highly selective and controllable release systems, enabling the efficient release of therapeutic agents in environments where the target biomarker is present. This strategy was later adapted to enable the release of gold nanoparticles with a distinct red color in the presence of glucose [41]. Recently, this approach was employed to develop CRISPR-responsive DNA hydrogels [42]. Another important subclass of hydrogels, known as stimuli-responsive colorimetric hydrogels, enables the quick visual detection of various substances. These hydrogels play a crucial role in applications such as detecting chemical warfare agents [155] and fumonisin B_1_ in food samples [156], among others.

#### 4.1.2. Drug Delivery

As for the drug delivery applications, a significant study by Kang et al. reported the creation of an injectable, thermoresponsive hydrogel nanocomposite for managing glioblastoma multiforme (GBM) post surgery [157]. This nanocomposite combines drug-loaded micelles with ferrimagnetic iron oxide nanocubes (wFIONs), which, when injected at the tumor resection site, form a gel at body temperature, establishing a stable intracortical depot for targeted drug release. The micelles are designed to deliver the drug directly to any remaining GBM cells, reducing early dispersion, while the wFIONs, when exposed to an alternating magnetic field, promote enhanced drug penetration. In tests with an orthotopic GBM mouse model, this hydrogel nanocomposite effectively slowed tumor growth and extended survival, underscoring its potential as a post-surgical GBM treatment. Lin et al. developed a chitosan-based micellar self-healing hydrogel (CM hydrogel) specifically designed to aid brain tissue regeneration after an intracerebral hemorrhage (ICH) stroke [158]. Composed of phenolic chitosan (PC) and a micellar crosslinker (DPF), this hydrogel mimics the mechanical characteristics of brain tissue and delivers two model drugs with staggered release patterns to ICH-affected rats, aiding in behavioral recovery and restoring brain movement balance. The CM hydrogel represents a promising new treatment for ICH stroke by promoting both neurogenesis and angiogenesis. Meanwhile, Wang et al. introduced an injectable, self-reinforcing nanogel-encapsulated hydrogel (NG@antagomir-21) loaded with blueantagomir-21 for gene therapy aimed at repairing degenerated nucleus pulposus [159]. This hydrogel provides essential mechanical support and preserves spinal segment stability. To enhance the efficacy of chemotherapy and alleviate the pain associated with colorectal cancer, a dual-drug delivery system (DDDS) was developed by Sheng et al. [160]. This system incorporates methotrexate (MTX)-loaded calcium carbonate (CaCO_3_/MTX) and aspirin (ASP) co-entrapped within hydrogels made of alginate and sodium carboxymethyl cellulose, crosslinked with Ca^2^⁺. The hydrogels protect MTX from premature absorption in the stomach and small intestine, ensuring its targeted release in the colorectum for maximum therapeutic effect. Notably, the DDDS achieves dual pH-responsive drug delivery. Given the pH variations in the small intestine and colorectum, the system enables the precise and responsive release of ASP and MTX at these respective sites. This innovation holds significant promise for advancing medical science and pharmaceutics, offering a targeted and efficient approach to colorectal cancer treatment. Tian et al. introduced an injectable nanostructured hydrogel designed for near-infrared-triggered drug release, combining photothermal and endocrine therapies for the synergistic treatment of endometriosis [161]. Due to the deep penetration of near-infrared light, the prepared hydrogel exhibited a significant temperature increase, enabling efficient photothermal therapy. Furthermore, elevated local temperatures enhanced the diffusion and penetration of letrozole, resulting in an excellent therapeutic effect in vivo. Importantly, both in vivo and in vitro tests demonstrated the nanocomposite hydrogel’s capability for endocrine-photothermal synergistic therapy, as well as its biocompatibility, highlighting its potential for advanced therapeutic applications. Additionally, Li et al. developed a pH-sensitive injectable hydrogel based on the octapeptide FOE, which breaks down within the tumor microenvironment. This degradation process promotes doxorubicin uptake by cancer cells through structural changes, offering potential improvements for the clinical use of anti-cancer drugs [162].

#### 4.1.3. Wound Healing and Tissue Engineering

**Figure 9:**
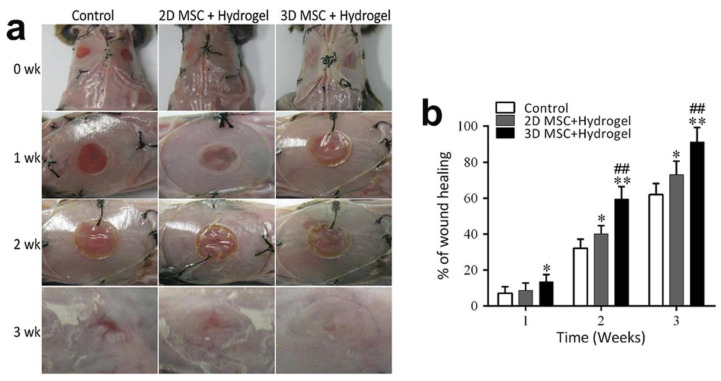
Wound-healing acceleration with 3D MSC spheroid-encapsulated hydrogel [165]. (**a**) Representative images of wounds in db/db mice treated with control (2D MSC without hydrogel), 2D MSC + hydrogel, and 3D MSC + hydrogel at weeks 0, 1, 2, and 3. (**b**) Wound closure areas were measured at weeks 1, 2, and 3. * *p* < 0.05, ** *p* < 0.01 versus control; ## *p* < 0.01 versus 2D MSC + hydrogel group.

Chronic wounds often contain biofilm-producing bacteria and experience high oxidative stress levels. Many current dressings for chronic wound healing require additional treatments, such as photothermal irradiation, or leave behind significant unwanted residues. Thermo-sensitive hydrogels, such as the polydopamine-modified triblock copolymer hydrogel (PDA/P2), represent a promising approach for bacteria-infected wound healing by combining sol–gel transition properties at body temperature (34–38 °C) with reactive oxygen species (ROS)-scavenging and antibacterial capabilities [163]. The ROS-scavenging ability of PDA/P2, demonstrated through DPPH and ABTS assays and intracellular ROS reduction in RAW264.7 cells, alleviates oxidative stress, while the encapsulation of silver nanoparticles (PDA/P2–4@Ag) provides broad-spectrum antibacterial activity against E. coli and S. aureus. In vivo studies using an S. aureus-infected rat model revealed the hydrogel’s ability to inhibit bacterial growth, reduce inflammation, and promote angiogenesis and collagen deposition, accelerating wound healing. These findings highlight the potential of PDA/P2-based hydrogels as multifunctional, temperature-sensitive wound dressings for advanced wound care applications. Thermogels, which undergo a phase change at physiological temperatures, have also been effectively utilized for managing diabetic wounds. For example, Jiang et al. [164] developed a temperature-responsive hydrogel patch composed of polymeric gallic acid (PGA) and gelatin methacryloyl (GelMA) for diabetic wound treatment. The PGA-GelMA hydrogel exhibits temperature-triggered adhesion and detachment properties due to its high density of non-covalent bonds. At body temperature (37 °C), the GelMA chains break down to form a soft hydrogel that adheres easily to the skin surface. This process exposes reactive motifs, such as amino and carboxyl groups, enabling the hydrogel to form multiple interfacial bonds and exhibit strong adhesion. Upon cooling with an ice pack, the GelMA chains re-entangle through intermolecular hydrogen bonding, restricting chain movement and breaking the adhesive bonds. In vivo experiments demonstrated the hydrogel’s biocompatibility, offering skin-friendly, nonallergenic adhesion, and nondestructive separation, making it an excellent candidate for diabetic wound care. Similarly, Yang et al. [165] proposed an innovative therapeutic approach utilizing injectable heat-sensitive hydrogels composed of chitosan, collagen, and β-glycerophosphate, combined with 3D mesenchymal stem cell (MSC) spheroids, to enhance wound healing in a diabetic mouse model. This strategy leverages the thermosensitive properties of the hydrogel for in situ gelation at body temperature, creating an optimal environment for MSC spheroids to promote tissue regeneration and accelerate healing in diabetic wounds (see Figure 9).

Due to the complexity of wound repair, effective treatment must be complemented by real-time, remote monitoring. To address this, Jiang et al. [166] developed a smart hydrogel wound dressing with conductive, temperature-responsive, antibacterial, and biocompatible properties. The dressing integrates polyacrylic acid (PAA)-grafted poly(*N*-isopropylacrylamide) as a temperature-responsive matrix, polyacrylamide for enhanced mechanical strength via semi-penetrating polymer networks, and silver nanowires for antibacterial and conductive functions. Equipped with a Bluetooth module, the hydrogel wirelessly transmits temperature data to a smart device, enabling real-time infection monitoring. This innovative design demonstrates the potential of smart hydrogel dressings to revolutionize wound care and diagnostic strategies.

### 4.2. Environmental Applications

#### 4.2.1. Detection and Removal of Heavy Metals

Heavy metals with a density greater than 4.5 g/cm^3^, such as Hg^2^⁺, Zn^2^⁺, Cd^2^⁺, Pb^2^⁺, and Cu^2^⁺, are common pollutants [151]. These metals exhibit properties such as concealment, hysteresis, accumulation, and resistance to degradation. They interact strongly with proteins and enzymes, leading to accumulation in various organs and posing significant risks to human health [167]. Consequently, the detection and removal of heavy metals from the environment and polluted water are of critical importance [168]. Zhang et al. developed a hydrophilic semi-IPN fluorescent polyHEAA hydrogel chemosensor for hazardous mercury (Hg^2^⁺) detection, addressing challenges posed by bacterial contamination in real-world samples [44]. Fabricated via UV polymerization, the hydrogel integrates a fluorescent polymer (PA-NDBCB) with a polyHEAA network, creating a robust semi-IPN structure with enhanced mechanical properties. The hydrogel detects Hg^2^⁺ ions through a highly efficient cyclization reaction with thiourea moieties, producing a visible “green-to-blue” fluorescence color change. Its hydrophilic, porous structure enables ultrafast, sensitive, and selective detection with a detection limit of 0.067 μM, even in complex aqueous systems. Remarkably, the hydrogel retains its fluorescence and detection capabilities under long-term coculture with E. coli, highlighting its potential for practical applications in mercury detection in real-world environments. Zhao et al. developed a solar-driven hydrogel (DCN) composed of photothermal polydopamine (PDA), linear chitosan (CTS), and thermoresponsive poly(*N*-isopropyl acrylamide) (PNIPAM) to remove heavy metal ions from contaminated water [169]. The DCN undergoes a hydrophilic/hydrophobic phase transition upon reaching its volume phase transition temperature (VPTT) under solar energy, achieving this in just 11 min under simulated sunlight. The hydrogel features an interpenetrating porous structure, high elasticity (withstanding up to 80% compressive deformation), and a low water contact angle of 13°, enhancing resistance to oil erosion. It demonstrated excellent selective adsorption for Pb^2^⁺ and Cu^2^⁺, with maximum adsorption capacities of 42.63 mg/g and 38.55 mg/g, respectively. Additionally, the phase transition significantly improved the hydrogel’s resistance to external interference and minimized heavy metal ion loss, showcasing its potential for effective water purification. Tohamy et al. developed highly swelling, stimuli-responsive hydrogels for the efficient adsorption of inorganic pollutants, particularly Cr(VI) [170]. These hydrogels were synthesized by grafting acrylamide (AM) and 3-sulfopropyl acrylate (SPA) onto hydroxypropyl methyl cellulose (HPMC), a cost-effective, hydrophilic natural polymer, through radical polymerization and crosslinking. Hydrogels with a higher SPA content (AM/SPA = 0.5) exhibited exceptional swelling behavior (equilibrium swelling ratio of 12,100%), rapid response to pH and temperature, and fast swelling kinetics, but with a lower modulus. Hydrogels with higher AM content (AM/SPA = 1 and 2) showed greater mechanical strength but more moderate pH and temperature responses. Cr(VI) adsorption tests demonstrated high efficiency, with 90–96% removal in a single step. Hydrogels with AM/SPA ratios of 0.5 and 1 showed strong potential for repeated use, as they could be regenerated via pH adjustments. Zheng et al. developed lignin-based dual-functional hydrogels for agrochemical delivery and heavy metal ion remediation in soil [171]. These hydrogels, synthesized through free-radical copolymerization, incorporate agrochemicals like 3-indoleacetic acid (IAC) and 2,4-dichlorophenoxyacetic acid (DCP), which are gradually released via ester bond cleavage to enhance crop growth and yield. The system effectively regulated lettuce growth, confirming its agricultural potential. Additionally, the hydrogels’ metal-chelating groups (COOH, phenolic OH, tertiary amine) enable the adsorption of heavy metals like Cu(II) (>380 mg/g) and Pb(II) (>60 mg/g), supporting soil remediation and reducing plant toxicity, making them a promising tool for sustainable agriculture.

#### 4.2.2. Removal of Phosphate

Dai et al. developed a self-crosslinked composite hydrogel using chitosan (CS) and cationic guar gum (CGG) via imine and acetal chemistry [172]. The CS/CGG hydrogel is thermally and pH-responsive, injectable, and adhesive and exhibits good compressive strength. It effectively removes phosphate from wastewater through multilayered adsorption, modeled by the Freundlich isotherm. Interestingly, after phosphate adsorption, the hydrogels were converted into N,P-doped carbon aerogels with excellent supercapacitor electrode performance, achieving a specific capacitance of 302.2 ± 4.9 F/g and stable cycling over 5000 charge/discharge cycles.

### 4.3. Soft Robotics, Actuators, and Other Emerging Applications

#### 4.3.1. Soft Robotics and Actuators

**Figure 10:**
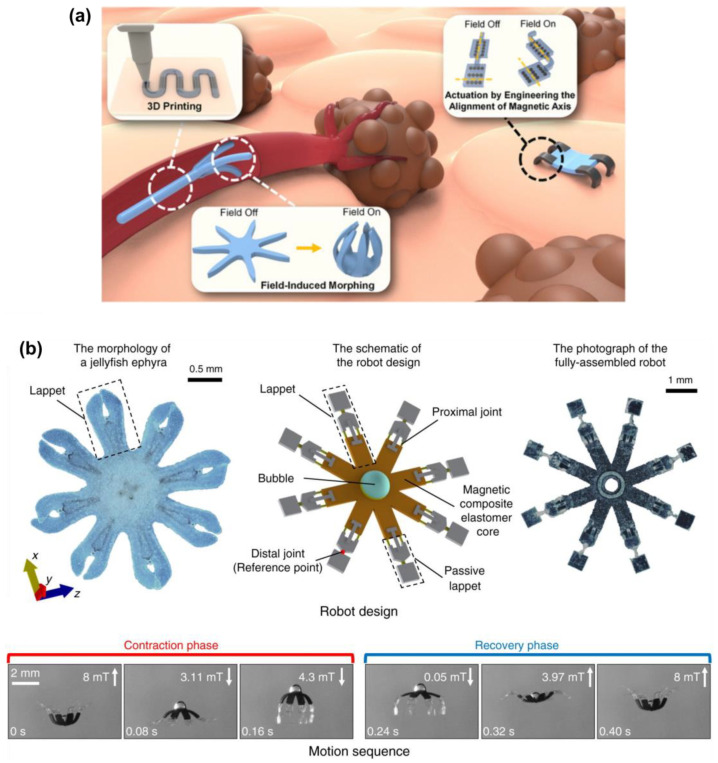
(**a**) Schematic of magnetically steered swimming and terrestrial robots targeting tumors [82]. (**b**) Design and swimming behavior of a jellyfish-inspired soft millirobot [174]. The arrows indicate the directions of the external magnetic field.

Soft robotics, inspired by biomimetic design, has emerged as a transformative technology bridging humans, robots, and physical labor [82,173]. Unlike conventional rigid robots, which rely on complex feedback systems for tasks like grasping fragile objects, soft robots use flexible, stretchable materials to achieve adaptive movements. Mimicking natural mechanisms, such as an octopus’s tentacles or a chameleon’s tongue, soft robotics enable intuitive actions like gripping, navigating tight spaces, and projecting objects, made possible by robust elastomers and hydrogels that dynamically change shape and dimensions. Figure 10a presents a schematic of magnetically steered swimming and terrestrial robots targeting tumors [82], while Figure 10b showcases the implementation of a multifunctional soft-bodied jellyfish-inspired swimmer [174]. This illustrates both the fundamental principle and the practical implementation of a soft robot.

An in-depth overview of hydrogel soft robotics capable of responding to different stimuli, including temperature, chemical stimuli, light, and electrical stimuli, can be found elsewhere [175,176]. Hydrogel actuators powered by chemical fuels face challenges due to waste accumulation, hindering their reusability. A recent study by Zhao et al. introduced ionic fuel-powered soft robotics using programmable, waste-free, salt-responsive hydrogel actuators [177]. Janus bilayer hydrogels, capable of swelling and shrinking, were developed and tested for mechanical properties, fuel efficiency, and structural integrity. The actuators exhibited adjustable bending and consistent cyclic performance with fuel replenishment and enabled the design of biomimetic soft robots with spatiotemporally controllable functions, demonstrating their potential for advanced soft robotics applications. Liu et al. [178] recently constructed the first self-sensing remote interaction system integrating gradient hydrogel actuators and robotic hands A bioinspired self-sensing gradient hydrogel was developed using a wettability-based method with MoO₂ nanosheet precipitation. The hydrogel demonstrated ultrafast thermoresponsive actuation, exceptional photothermal efficiency, and high sensing capability. The fabrication of dual-responsive soft robots often involves complex designs, but a recent study by Siebenmorgen et al. presented a minimally designed solution using pNIPAAm and ferromagnetic particles [179]. Free radical polymerization ensures the uniform embedding of magnetic particles within the polymer network. The robot integrates magnetic shape programming and thermal responsiveness, enabling versatile tasks such as shaping, locomotion, pick-and-place, and object release using independent triggers. Magnetic actuation immobilizes the robot in a gripping state, while a temperature increase transitions it from swollen to collapsed, allowing secure configurations during magnetic movements. This straightforward approach achieves precise dual-stimuli control for soft robotics. López-Díaz et al. recently developed an electroactive hydrogel based on a cationic network that enables bending motion even outside aqueous environments [180,181]. By leveraging the water retained within the hydrogel matrix, this innovative design expands the functionality of electroactive hydrogels, allowing them to generate mechanical responses in non-aqueous settings. This advancement opens new possibilities for soft robotics and actuation systems in diverse environmental conditions. Combining stimuli-responsive hydrogels with plasmonic nanostructures offers unique advantages for soft robotics and actuators, enabling functionalities beyond what each component achieves individually [182]. Plasmonic nanomaterials, widely used in light harvesting and molecular sensing, can now be dynamically tuned when integrated with responsive hydrogels. This synergy allows for rapid mechanical actuation and nanoscale light confinement with resonant intensity amplification. Recent advancements highlight their application in plasmonic biosensing, plasmon-enhanced optical spectroscopy, optically driven soft actuators, and light-fueled micromachines, offering innovative solutions for systems mimicking biological environments.

#### 4.3.2. Other Emerging Applications

**Figure 11:**
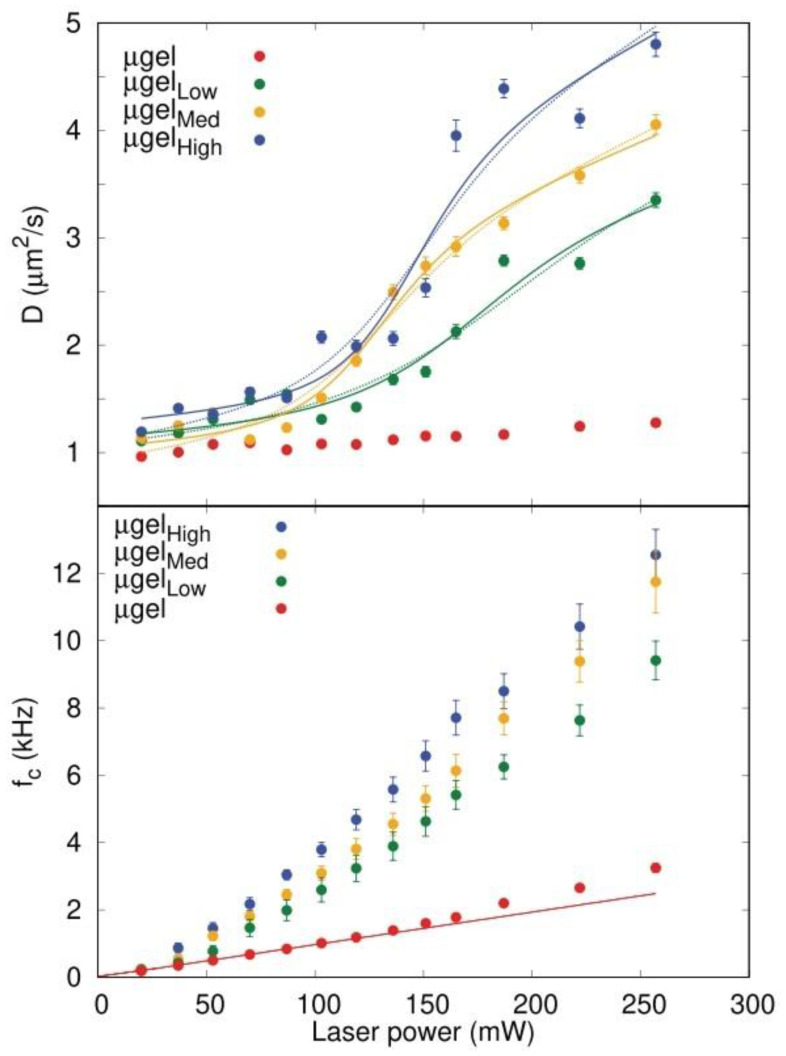
The averaged diffusion coefficient *D* and corner frequency *f*_c_ values of 12 microgels per sample are presented for samples with different amounts of iron oxide nanocubes [19]. The dashed and solid lines represent linear and Gaussian-like fittings, respectively, enabling the calibration of temperature corresponding to each laser power.

Unconventional yet emerging applications of stimuli-responsive hydrogels include the use of hydrogel actuators for cell mechanical stimulation, enabling the exploration of mechanotransduction processes. Recently, the magnetic actuation of magnetically responsive hydrogel structures was demonstrated for investigating cell mechanotransduction [86,87]. This approach enables precise dynamic and static actuation through external magnetic fields, allowing for the customizable control of cell growth, morphogenesis, and behavior at the molecular level. In overall, this approach holds significant potential for precise and localized cell actuation. Another interesting yet still not fully investigated possible application of the thermoresponsive hydrogels as local micro-thermometers was proposed by Fernandez-Rodriguez et al. [19]. The self-heating iron nanocube-decorated microgels prepared in this study exhibited a discontinuous transition when a single microgel was isolated in an optical trap, whereas a sigmoidal-like continuous transition was observed when averaged across multiple microgels. The continuous behavior allows precise temperature tuning at each laser power density (see Figure 11), making them suitable for applications as local micro-heaters and micro-thermometers. As this capability is highly sought after in nanomedicine and materials science, future research could help transition it into practical applications.

In addition to combining stimuli-responsive hydrogels with plasmonic and magnetic structures, such as nanoparticles, as discussed above, their performance can also be enhanced or modified by integrating them with carbonaceous nanoparticles [183], stiff inclusions [184], other polymers [94], biologically active components [185,186], mesoporous silica nanoparticles [187], and various other inorganic particles [188].

## 5. Current Challenges and Future Perspectives

**Figure 12:**
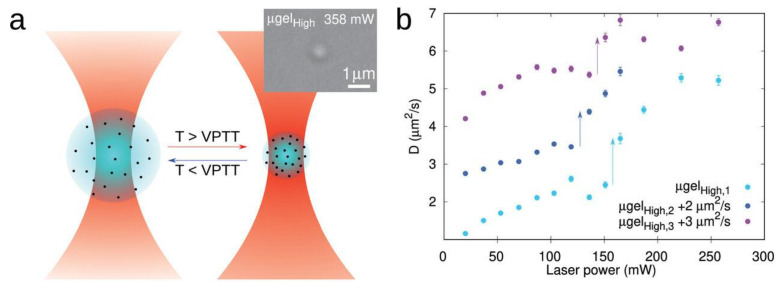
(**a**) Schematic representation of an optically trapped microgel decorated with iron oxide nanocubes, below and above the VPTT. The inset shows the trapped microgel captured with a CMOS camera. (**b**) Diffusivity *D* of individual microgels as functions of laser power *P*. The *D* plots are vertically offset for clarity, as indicated in the legends. Arrows mark the discontinuous transitions for each microgel [19].

Despite significant advancements in the field of stimuli-responsive hydrogels, several challenges remain, even at the fundamental level of understanding their behavior. For instance, microgels exhibiting thermoresponsiveness undergo a transformation at a critical temperature known as the volume phase transition temperature (VPTT). However, whether this transformation is smooth or discontinuous remains a topic of ongoing debate. The thermoresponsive transition of microgels is commonly described as following a sigmoidal smooth curve [189]. For microgels with sizes in the range of a few tens of microns, this transition is often considered continuous [190]. Recent studies have utilized optical tweezers to investigate the VPTT of thermoresponsive microgels at the single-microgel level under external heating conditions [191,192]. However, findings remain inconsistent. One study reported a discontinuous transition [191], while another observed a smooth, sigmoidal transition [192]. Additionally, a separate investigation by the same authors reported a discontinuous transition for pH-responsive microgels in response to pH changes [193], leaving the conclusions regarding these transitions inconclusive. In a recent study [19], Fernandez-Rodriguez et al. demonstrated that a single iron nanocube-decorated microgel undergoes a discontinuous volume phase transition beyond a specific laser power (see Figure 12), whereas the typical continuous sigmoidal-like behavior is observed when results are averaged across multiple microgels. This example highlights the necessity for a more systematic investigation of phase transitions in stimuli-responsive hydrogels and the organization of findings to establish a clearer understanding.

Another example is the incomplete understanding of hydrogel behavior when an external stimulus is switched off. Auer et al. [194] reported abrupt changes in the recorded signal upon beam deactivation, which can be linked to the Kovacs memory effect [195]. This effect occurs when nonequilibrium cooling and heating processes at the nanoscale follow unexpected relaxation paths due to memory effects, significantly altering the anticipated equilibration trajectory. Specifically, during thermalization, a sudden change in the temperature can lead to a nonmonotonic evolution of the system’s energy. This underscores the need for systematic studies of such processes in stimuli-responsive hydrogel-based systems and their generalization within the framework of nonequilibrium thermodynamics.

Regarding the mechanical properties of hydrogels, inconsistencies in measured data remain a challenge. For example, a comparison of the existing literature on the mechanical properties of agar and polyacrylamide gels highlights the uncertainties and difficulties associated with gel mechanical characterization [98]. Future research is needed to address these uncertainties and improve the reliability of mechanical characterization for hydrogels.

Stimuli-responsive hydrogels present immense potential across various fields, but significant practical challenges remain to transition these materials from the laboratory to practical applications. Key concerns include biocompatibility, biodegradability, scalability, and economic feasibility. Biocompatibility issues stem from the potential cytotoxicity of some hydrogel components or their degradation products, while biodegradability is often dependent on the precise chemical composition, making it challenging to balance functionality and environmental sustainability. Scalability is hindered by the complex synthesis processes of stimuli-responsive hydrogels, which often require controlled conditions, multi-step reactions, or expensive raw materials, limiting their cost-effectiveness and large-scale production. Material performance under operational conditions also presents challenges. Limitations in durability, response time, and reliability under repeated or prolonged stimulus exposure affect their long-term usability. For instance, repeated swelling and deswelling cycles can cause mechanical fatigue or structural degradation, undermining performance in applications such as drug delivery or actuation. Additionally, hydrogel response times may be too slow for dynamic applications, particularly in soft robotics or biosensing, necessitating material innovations to enhance response speed without compromising other properties. Future research should focus on addressing these issues by developing more sustainable and cost-effective production techniques, such as green synthesis methods and bio-derived polymers. Enhancing responsiveness through advanced material design, such as incorporating nanostructures or optimizing polymer architecture, can further expand their applicability. Expanding biocompatibility by fine-tuning degradation rates and ensuring safe byproducts is critical for medical and environmental applications.

Emerging fields, including personalized medicine and advanced environmental technologies, offer new opportunities for these materials. For instance, stimuli-responsive hydrogels could enable precision drug delivery systems that respond to patient-specific biomarkers, enhancing therapeutic efficacy while minimizing side effects. In environmental applications, hydrogels with selective adsorption and degradation properties could transform pollutant removal, addressing challenges such as heavy metal or microplastic contamination. Additionally, we foresee increased research focus on cell mechanotransduction enabled by stimuli-responsive hydrogel actuators, including magnetic-, thermo-, and other stimuli-based systems.

Achieving these goals requires systematic investigations and regular comprehensive reviews. Detailed studies on mechanical properties, phase transition behavior, and interactions under multi-stimuli conditions are required to inform the design of next-generation hydrogels. Establishing robust frameworks for characterization and understanding the fundamental science behind stimulus interactions will be pivotal. Addressing these scientific and technical gaps will accelerate the integration of stimuli-responsive hydrogels into practical solutions, ultimately bringing their transformative potential closer to societal use.

## 6. Conclusions

Stimuli-responsive hydrogels represent a rapidly advancing field of research, with significant potential to address critical societal challenges. In this review, we summarized fundamental concepts and provided an overview of applications across established and emerging areas. Hydrogels were classified by their response mechanisms, with key properties such as swelling kinetics, mechanical behavior, and biocompatibility/biodegradability examined. Advancements in design, synthesis, and structural engineering were also discussed, with applications highlighted in biomedical fields (biosensing, drug delivery, wound healing, and tissue engineering), environmental solutions (heavy metal and phosphate removal), and soft robotics and actuation.

Emerging applications, including local micro-thermometers and cell mechanotransduction, were reviewed to illustrate the versatility of these materials. However, the field remains dynamic, with unresolved challenges such as scalability, durability, response time, and economic feasibility requiring attention. Fundamental questions, including mechanical properties, phase transition behavior, and multi-stimuli interactions, also need further investigation to fully realize the potential of these materials.

While it was not feasible to address every advancement in this expansive field, we hope this review serves as a comprehensive starting point, inspiring further research and innovation in stimuli-responsive hydrogels and their transformative applications.

## Data Availability

No new data were created or analyzed in this study.

## References

[1] Ahmed E.M. (2015). Hydrogel: Preparation, characterization, and applications: A review. J. Adv. Res..

[2] Picchioni F., Muljana H. (2018). Hydrogels based on dynamic covalent and non covalent bonds: A chemistry perspective. Gels.

[3] Perera M.M., Ayres N. (2020). Dynamic covalent bonds in self-healing, shape memory, and controllable stiffness hydrogels. Polym. Chem..

[4] Ye J., Fu S., Zhou S., Li M., Li K., Sun W., Zhai Y. (2020). Advances in hydrogels based on dynamic covalent bonding and prospects for its biomedical application. Eur. Polym. J..

[5] Shahi S., Roghani-Mamaqani H., Hoogenboom R., Talebi S., Mardani H. (2022). Stimuli-responsive covalent adaptable hydrogels based on homolytic bond dissociation and chain transfer reactions. Chem. Mater..

[6] Han Y., Cao Y., Lei H. (2022). Dynamic covalent hydrogels: Strong yet dynamic. Gels.

[7] Wu M., Han L., Yan B., Zeng H. (2023). Self-healing hydrogels based on reversible noncovalent and dynamic covalent interactions: A short review. Supramol. Mater..

[8] Wichterle O., Lím D. (1960). Hydrophilic gels for biological use. Nature.

[9] Chen W.-H., Liao W.-C., Sohn Y.S., Fadeev M., Cecconello A., Nechushtai R., Willner I. (2018). Stimuli-responsive nucleic acid-based polyacrylamide hydrogel-coated metal–organic framework nanoparticles for controlled drug release. Adv. Funct. Mater..

[10] Del Valle L.J., Díaz A., Puiggalí J. (2017). Hydrogels for biomedical applications: Cellulose, chitosan, and protein/peptide derivatives. Gels.

[11] Zhu T., Mao J., Cheng Y., Liu H., Lv L., Ge M., Li S., Huang J., Chen Z., Li H. (2019). Recent progress of polysaccharide-based hydrogel interfaces for wound healing and tissue engineering. Adv. Mater. Interfaces.

[12] Liu X., Zhang Q., Qiao Y., Duan L., Gao G. (2018). Anti-fatigue adhesive and tough hydrogels regulated by adenine and uracil. Polym. Chem..

[13] Amaral A.J.R., Emamzadeha M., Pasparakis G. (2018). Transiently malleable multi-healable hydrogel nanocomposites based on responsive boronic acid copolymers. Polym. Chem..

[14] Yu H., Wang Y., Yang H., Peng K., Zhang X. (2017). Injectable self-healing hydrogels formed via thiol/disulfide exchange of thiol functionalized F127 and dithiolane modified PEG. J. Mater. Chem. B.

[15] Guo M., Pitet L.M., Wyss H.M., Vos M., Dankers P.Y.W., Meijer E.W. (2014). Tough stimuli-responsive supramolecular hydrogels with hydrogen-bonding network junctions. J. Am. Chem. Soc..

[16] Zhang K., Yuan W., Wei K., Yang B., Chen X., Li Z., Zhang Z., Bian L. (2019). Highly dynamic nanocomposite hydrogels self-assembled by metal ion-ligand coordination. Small.

[17] Hamzah Y.B., Hashim S., Rahman W.A.W.A. (2017). Synthesis of polymeric nano/microgels: A review. J. Polym. Res..

[18] Roy A., Manna K., Pal S. (2022). Recent advances in various stimuli-responsive hydrogels: From synthetic designs to emerging healthcare applications. Mater. Chem. Front..

[19] Fernandez-Rodriguez M.A., Orozco-Barrera S., Sun W., Gámez F., Caro C., García-Martín M.L., Rica R.A. (2023). Hot Brownian motion of thermoresponsive microgels in optical tweezers shows discontinuous volume phase transition and bistability. Small.

[20] Heskins M., Guillet J.E. (1968). Solution properties of poly(N-isopropylacrylamide). J. Macromol. Sci. A.

[21] Beines P.W., Klosterkamp I., Menges B., Jonas U., Knoll W. (2007). Responsive thin hydrogel layers from photo-cross-linkable poly(N-isopropylacrylamide) terpolymers. Langmuir.

[22] Eyigor A., Bahadori F., Yenigun V.B., Eroglu M.S. (2018). Beta-Glucan based temperature responsive hydrogels for 5-ASA delivery. Carbohydr. Polym..

[23] Dai S., Ravi P., Tam K.C. (2008). pH-Responsive polymers: Synthesis, properties and applications. Soft Matter.

[24] Huang J., Jiang X. (2018). Injectable and degradable pH-responsive hydrogels via spontaneous amino–yne click reaction. ACS Appl. Mater. Interfaces.

[25] Vegad U., Patel M., Khunt D., Zupančič O., Chauhan S., Paudel A. (2023). pH stimuli-responsive hydrogels from non-cellulosic biopolymers for drug delivery. Front. Bioeng. Biotechnol..

[26] Tomatsu I., Peng K., Kros A. (2011). Photoresponsive hydrogels for biomedical applications. Adv. Drug Deliv. Rev..

[27] Li L., Scheiger J.M., Levkin P.A. (2019). Design and applications of photoresponsive hydrogels. Adv. Mater..

[28] Jiang H., Fan L., Yan S., Li F., Li H., Tang J. (2019). Tough and electro-responsive hydrogel actuators with bidirectional bending behavior. Nanoscale.

[29] Carayon I., Gaubert A., Mouslia Y., Philippe B. (2020). Electro-responsive hydrogels: Macromolecular and supramolecular approaches in the biomedical field. Biomater. Sci..

[30] Zhou X., Zhang N., Kandalai S., Li H., Hossain F., Zhang S., Zhu J., Zhang J., Cui J., Zheng Q. (2023). Dynamic and wearable electro-responsive hydrogel with robust mechanical properties for drug release. ACS Appl. Mater. Interfaces.

[31] Li Z., Li Y., Chen C., Cheng Y. (2021). Magnetic-responsive hydrogels: From strategic design to biomedical applications. J. Control. Release.

[32] Fragal E.H., Fragal V.H., Silva E.P., Paulino A.T., da Silva Filho E.C., Mauricio M.R., Silva R., Rubira A.F., Muniz E.C. (2022). Magnetic-responsive polysaccharide hydrogels as smart biomaterials: Synthesis, properties, and biomedical applications. Carbohydr. Polym..

[33] Gao F., Jiang H., Wang D., Wang S., Song W. (2024). Bio-inspired magnetic-responsive supramolecular-covalent semi-convertible hydrogel. Adv. Mater..

[34] Hou X., Li Y., Pan Y., Jin Y., Xiao H. (2018). Controlled release of agrochemicals and heavy metal ion capture dual-functional redox-responsive hydrogel for soil remediation. Chem. Commun..

[35] Boz R.K., Aydin D., Kocak S., Golba B., Sanyal R., Sanyal A. (2022). Redox-responsive hydrogels for tunable and “on-demand” release of biomacromolecules. Bioconjugate Chem..

[36] Ambrožič R., Krühne U., Plazl I. (2024). Microfluidics with redox-responsive hydrogels for on-demand BPA degradation. Chem. Eng. J..

[37] Cherri M., Stergiou P.S., Ahmadian Z., Povolotsky T.L., Thongrom B., Fan X., Mohammadifar E., Haag R. (2024). Redox-responsive hydrogels loaded with an antibacterial peptide as controlled drug delivery for healing infectious wounds. Adv. Healthc. Mater..

[38] Ebrahimi R., Tarhande G., Rafiei S. (2012). The study of ultrasonic degradation of superabsorbent hydrogels. Org. Chem. Int..

[39] Zhou Y., Liu G., Guo S. (2022). Advances in ultrasound-responsive hydrogels for biomedical applications. J. Mater. Chem. B.

[40] Yang H., Liu H., Kang H., Tan W. (2008). Engineering target-responsive hydrogels based on aptamer−target interactions. J. Am. Chem. Soc..

[41] Ma Y., Mao Y., An Y., Tian T., Zhang H., Yan J., Zhu Z., Yang C.J. (2018). Target-responsive DNA hydrogel for non-enzymatic and visual detection of glucose. Analyst.

[42] Guo J., Zhu Y., Miao P. (2023). Nano-impact electrochemical biosensing based on a CRISPR-responsive DNA hydrogel. Nano Lett..

[43] Culver H.R., Clegg J.R., Peppas N.A. (2017). Analyte-responsive hydrogels: Intelligent materials for biosensing and drug delivery. Acc. Chem. Res..

[44] Zhang D., Yao Y., Wu J., Protsak I., Lu W., He X., Xiao S., Zhong M., Chen T., Yang J. (2019). Super hydrophilic semi-IPN fluorescent poly(N-(2-hydroxyethyl)acrylamide) hydrogel for ultrafast, selective, and long-term effective mercury(II) detection in a bacteria-laden system. ACS Appl. Bio Mater..

[45] Li J., Mooney D.J. (2016). Designing hydrogels for controlled drug delivery. Nat. Rev. Mater..

[46] Vigata M., Meinert C., Hutmacher D.W., Bock N. (2020). Hydrogels as drug delivery systems: A review of current characterization and evaluation techniques. Pharmaceutics.

[47] Nassar N., Kasapis S. (2023). Fundamental advances in hydrogels for the development of the next generation of smart delivery systems as biopharmaceuticals. Int. J. Pharm..

[48] Zhang D., Ren B., Zhang Y., Xu L., Huang Q., He Y., Li X., Wu J., Yang J., Chen Q. (2020). From design to applications of stimuli-responsive hydrogel strain sensors. J. Mater. Chem. B.

[49] Toma M., Jonas U., Mateescu A., Knoll W., Dostalek J. (2013). Active control of SPR by thermoresponsive hydrogels for biosensor applications. J. Phys. Chem. C.

[50] Han F., Wang T., Liu G., Liu H., Xie X., Wei Z., Li J., Jiang C., He Y., Xu F. (2022). Materials with tunable optical properties for wearable epidermal sensing in health monitoring. Adv. Mat..

[51] Van Vlierberghe S., Dubruel P., Schacht E. (2011). Biopolymer-based hydrogels as scaffolds for tissue engineering applications: A review. Biomacromolecules.

[52] Zhao C., Qazvini N.T., Sadati M., Zeng Z., Huang S., De La Lastra A.L., Zhang L., Feng Y., Liu W., Huang B. (2019). A pH-triggered, self-assembled, and bioprintable hybrid hydrogel scaffold for mesenchymal stem cell based bone tissue engineering. ACS Appl. Mater. Interfaces.

[53] Das D., Cha E., Lee S., Shin H., Noh I. (2020). Effects of molar ratios of two immiscible monomers toward development of an amphiphilic, highly stretchable, bioadhesive, self-healing copolymeric hydrogel and its mineral-active cellular behavior. Biomacromolecules.

[54] Roy A., Ray P.G., Manna K., Banerjee C., Dhara S., Pal S. (2021). Poly(N-vinyl imidazole) cross-linked β-cyclodextrin hydrogel for rapid hemostasis in severe renal arterial hemorrhagic model. Biomacromolecules.

[55] Löwenberg C., Balk M., Wischke C., Behl M., Lendlein A. (2017). Shape-memory hydrogels: Evolution of structural principles to enable shape switching of hydrophilic polymer networks. Acc. Chem. Res..

[56] Farrukh A., Nayab S. (2024). Shape memory hydrogels for biomedical applications. Gels.

[57] Kim Y.S., Liu M., Ishida Y., Ebina Y., Osada M., Sasaki T., Hikima T., Takata M., Aida T. (2015). Thermoresponsive actuation enabled by permittivity switching in an electrostatically anisotropic hydrogel. Nature Mater..

[58] Zheng W.J., An N., Yang J.H., Zhou J., Chen Y.M. (2015). Tough Al-alginate/poly(N-isopropylacrylamide) hydrogel with tunable LCST for soft robotics. ACS Appl. Mater. Interfaces.

[59] Klouda L., Mikos A.G. (2008). Thermoresponsive hydrogels in biomedical applications. Eur. J. Pharm. Biopharm..

[60] Junk M.J.N., Berger R., Jonas U. (2010). Atomic force spectroscopy of thermoresponsive photo-cross-linked hydrogel films. Langmuir.

[61] Le M., Huang W., Chen K.F., Lin C., Cai L., Zhang H., Jia Y.G. (2022). Upper critical solution temperature polymeric drug carriers. Chem. Eng. J..

[62] Salminen L., Karjalainen E., Aseyev V., Tenhu H. (2022). Phase separation of aqueous poly(diisopropylaminoethyl methacrylate) upon heating. Langmuir.

[63] He J., Zhou Q., Ge Z., Jiang S., Li J., Feng W., Yang H. (2024). pH-gated switch of LCST-UCST phase transition of hydrogels. Adv. Funct. Mat..

[64] Kim Y.-J., Matsunaga Y.T. (2017). Thermo-responsive polymers and their application as smart biomaterials. J. Mater. Chem. B.

[65] Zhang K., Xue K., Loh X.J. (2021). Thermo-responsive hydrogels: From recent progress to biomedical applications. Gels.

[66] Dou Q.Q., Liow S.S., Ye E., Lakshminarayanan R., Loh X.J. (2014). Biodegradable thermogelling polymers: Working towards clinical applications. Adv. Healthc. Mater..

[67] Liow S.S., Dou Q., Kai D., Karim A.A., Zhang K., Xu F., Loh S.J. (2016). Thermogels: In situ gelling biomaterial. ACS Biomater. Sci. Eng..

[68] Moon H.J., Ko D.Y., Park M.H., Joo M.K., Jeong B. (2012). Temperature-responsive compounds as in situ gelling biomedical materials. Chem. Soc. Rev..

[69] Jayakumar A., Jose V.K., Lee J.-M. (2020). Hydrogels for medical and environmental applications. Small Methods.

[70] Yu H., Gao R., Liu Y., Fu L., Zhou J., Li L. (2024). Stimulus-responsive hydrogels as drug delivery systems for inflammation targeted therapy. Adv. Sci..

[71] Feringa B.L., van Delden R.A., Koumura N., Geertsema E.M. (2000). Chiroptical molecular switches. Chem. Rev..

[72] Alfimov M.V., Fedorova O.A., Gromov S.P. (2003). Photoswitchable molecular receptors. J. Photochem. Photobiol. A Chem..

[73] Frachini E.C.G., Petri D.F.S. (2019). Magneto-responsive hydrogels: Preparation, characterization, biotechnological and environmental applications. J. Braz. Chem. Soc..

[74] Yu X., Yang R., Wu C., Liu B., Zhang W. (2022). The heating efficiency of magnetic nanoparticles under an alternating magnetic field. Sci. Rep..

[75] Myrovali E., Papadopoulos K., Charalampous G., Kesapidou P., Vourlias G., Kehagias T., Angelakeris M., Wiedwald U. (2023). Toward the separation of different heating mechanisms in magnetic particle hyperthermia. ACS Omega.

[76] Adedoyin A.A., Ekenseair A.K. (2018). Biomedical applications of magneto-responsive scaffolds. Nano Res..

[77] Ding H., Hao L., Mao H. (2024). Magneto-responsive biocomposites in wound healing: From characteristics to functions. J. Mater. Chem. B.

[78] Ganguly S., Margel S. (2021). Design of magnetic hydrogels for hyperthermia and drug delivery. Polymers.

[79] Ganguly S., Margel S. (2022). 3D printed magnetic polymer composite hydrogels for hyperthermia and magnetic field driven structural manipulation. Prog. Polym. Sci..

[80] Vítková L., Kazantseva N., Musilová L., Smolka P., Valášková K., Kocourková K., Humeník M., Minařík A., Humpolíček P., Mráček A. (2023). Magneto-responsive hyaluronan hydrogel for hyperthermia and bioprinting: Magnetic, rheological properties and biocompatibility. APL Bioeng..

[81] Tognato R., Armiento A.R., Bonfrate V., Levato R., Malda J., Alini M., Eglin D., Giancane G., Serra T. (2019). A stimuli-responsive nanocomposite for 3D anisotropic cell-guidance and magnetic soft robotics. Adv. Funct. Mat..

[82] Chung H.-J., Parsons A.M., Zheng L. (2021). Magnetically controlled soft robotics utilizing elastomers and gels in actuation: A review. Adv. Intell. Syst..

[83] Yarali E., Baniasadi M., Zolfagharian A., Chavoshi M., Arefi F., Hossain M., Bastola A., Ansari M., Foyouzat A., Dabbagh A. (2022). Magneto-/ electro-responsive polymers toward manufacturing, characterization, and biomedical/ soft robotic applications. Appl. Mater. Today.

[84] Khalid M.Y., Arif Z.U., Tariq A., Hossain M., Khan K.A., Umer R. (2024). 3D printing of magneto-active smart materials for advanced actuators and soft robotics applications. Eur. Polym. J..

[85] Heidarian P., Kouzani A.Z. (2023). A self-healing magneto-responsive nanocellulose ferrogel and flexible soft strain sensor. Int. J. Biol. Macromol..

[86] Goodrich R., Tai Y., Ye Z., Yin Y., Nam J. (2023). A magneto-responsive hydrogel system for the dynamic mechano-modulation of stem cell niche. Adv. Funct. Mat..

[87] Geid N., Husari A., Galli E., Leffler L., Tomakidi P., Rühe J. (2024). A gym for cells—Direct laser writing of magnetic multilayered micro actuators for mechanical stimulation of cells. Adv. Funct. Mat..

[88] Pourjavadi A., Heydarpour R., Tehrani Z.M. (2021). Multi-stimuli-responsive hydrogels and their medical applications. New J. Chem..

[89] Suryavanshi P., Mahajan S., Banerjee S.K., Seth K., Banerjee S. (2024). Synthesis and characterization of a pH/temperature-dual responsive hydrogel with promising biocompatibility features for stimuli-responsive 5-FU delivery. J. Mater. Chem. B.

[90] Rittikulsittichai S., Kolhatkar A.G., Sarangi S., Vorontsova M.A., Vekilov P.G., Brazdeikis A., Lee T.R. (2016). Multi-responsive hybrid particles: Thermo-, pH-, photo-, and magneto-responsive magnetic hydrogel cores with gold nanorod optical trigger. Nanoscale.

[91] Zhou X., Wang L., Xu Y., Du W., Cai X., Wang F., Ling Y., Chen H., Wang Z., Hu B. (2018). A pH and magnetic dual-response hydrogel for synergistic chemo-magnetic hyperthermia tumor therapy. RSC Adv..

[92] Liu X., Li B., Wang W., Zhang Y., Li H., Li Z. (2022). Multistimuli-responsive hydrogels with both anisotropic mechanical performance and anisotropic luminescent behavior. Chem. Eng. J..

[93] Morozov Y.M., Wiesner (née Diehl) F., Grün J.J., Pertiller M., Fossati S., Schmidt K., Quilis N.G., Gusenbauer C., Zbiral B., Toca-Herrera J.L. (2024). Microstructuring of thermoresponsive biofunctional hydrogels by multiphoton photocrosslinking. Adv. Funct. Mat..

[94] Osváth Z., Tóth T., Iván B. (2017). Sustained drug release by thermoresponsive sol–gel hybrid hydrogels of poly(N-isopropylacrylamide-co-3-(trimethoxysilyl)propyl methacrylate) copolymers. Macromol. Rapid Commun..

[95] Berens A.R., Hopfenberg H.B. (1978). Diffusion and relaxation in glassy polymer powders: 2. Separation of diffusion and relaxation parameters. Polymer.

[96] Anseth K.S., Bowman C.N., Brannon-Peppas L. (1996). Mechanical properties of hydrogels and their experimental determination. Biomaterials.

[97] Shaw M.T., MacKnight W.J. (2005). Introduction to Polymer Viscoelasticity.

[98] Oyen M.L. (2014). Mechanical characterisation of hydrogel materials. Int. Mater. Rev..

[99] Tokita M., Tanaka M. (1991). Friction coefficient of polymer networks of gels. J. Chem. Phys..

[100] Bailey M., Alunni-Cardinali M., Correa N., Caponi S., Holsgrove T., Barr H., Stone N., Winlove C.P., Fioretto D., Palombo F. (2020). Viscoelastic properties of biopolymer hydrogels determined by Brillouin spectroscopy: A probe of tissue micromechanics. Sci. Adv..

[101] Mahmodi H., Piloni A., Utama R.H., Kabakova I. (2021). Mechanical mapping of bioprinted hydrogel models by brillouin microscopy. Bioprinting.

[102] Kopač T. (2024). Mathematical model for characterization of temperature-responsive polymers: A study on the rheological behavior of gelatin and poly(N-isopropylacrylamide). Polym. Test..

[103] Khan Y., Bashir S., Hina M., Ramesh S., Ramesh K., Mujtaba M.A., Lahiri I., Ramesh S. (2020). Effect of charge density on the mechanical and electrochemical properties of poly (acrylic acid) hydrogel electrolytes based flexible supercapacitors. Mater. Today Commun..

[104] Zhao X., Chen X., Yuk H., Lin S., Liu X., Parada G. (2021). Soft materials by design: Unconventional polymer networks give extreme properties. Chem. Rev..

[105] Yang J., Chen Y., Zhao L., Zhang J., Luo H. (2023). Constructions and properties of physically cross-linked hydrogels based on natural polymers. Polym. Rev..

[106] Zhang Y., Wang S., Tian Y., Chen L., Du Y., Su G., Hu Y. (2023). Multi-physically cross-linked hydrogels for flexible sensors with high strength and self-healing properties. Polymers.

[107] Zhao D., Rong Y., Li D., He C., Chen X. (2023). Thermo-induced physically crosslinked polypeptide-based block copolymer hydrogels for biomedical applications. Regen. Biomater..

[108] Maiz-Fernández S., Pérez-Álvarez L., Silván U., Vilas-Vilela J.L., Lanceros-Méndez S. (2022). pH-induced 3D printable chitosan hydrogels for soft actuation. Polymers.

[109] Bashir S., Hina M., Iqbal J., Rajpar A.H., Mujtaba M.A., Alghamdi N.A., Wageh S., Ramesh K., Ramesh S. (2020). Fundamental concepts of hydrogels: Synthesis, properties, and their applications. Polymers.

[110] Zhang Y., Hu Q., Sun W., Sun T., Wang T., Liu H., Tong Z. (2024). pH-induced mechanical transition in polyion complex hydrogels: Shifting from ionic association to hydrophobic association. Macromolecules.

[111] Dobrynin A.V., Colby R.H., Rubinstein M. (2004). Polyampholytes. J. Polym. Sci. Part B Polym. Phys..

[112] Sun T.L., Kurokawa T., Kuroda S., Ihsan A.B., Akasaki T., Sato K., Haque M.A., Nakajima T., Gong J.P. (2013). Physical hydrogels composed of polyampholytes demonstrate high toughness and viscoelasticity. Nat. Mater..

[113] Chen K., Hu Y., Wang F., Liu M., Liu P., Li C., Yu Y., Xiao X., Feng Q. (2022). Ultra-stretchable, adhesive, and self-healing MXene/polyampholytes hydrogel as flexible and wearable epidermal sensors. Colloids Surf. A Physicochem. Eng. Asp..

[114] Liu X., He X., Yang B., Lai L., Chen N., Hu J., Lu Q. (2021). Dual physically cross-linked hydrogels incorporating hydrophobic interactions with promising repairability and ultrahigh elongation. Adv. Funct. Mat..

[115] Jiang P., Lin P., Yang C., Qin H., Wang X., Zhou F. (2020). 3D printing of dual-physical cross-linking hydrogel with ultrahigh strength and toughness. Chem. Mater..

[116] Wang G., Liu Y., Zu B., Lei D., Guo Y., Wang M., Dou X. (2023). Reversible adhesive hydrogel with enhanced sampling efficiency boosted by hydrogen bond and van der Waals force for visualized detection. Chem. Engineer. J..

[117] Ma X., Tian H. (2014). Stimuli-responsive supramolecular polymers in aqueous solution. Acc. Chem. Res..

[118] Shigemitsu H., Hamachi I. (2017). Design strategies of stimuli-responsive supramolecular hydrogels relying on structural analyses and cell-mimicking approaches. Acc. Chem. Res..

[119] Nandi N., Gayen K., Ghosh S., Bhunia D., Kirkham S., Sen S.K., Ghosh S., Hamley I.W., Banerjee A. (2017). Amphiphilic peptide-based supramolecular, noncytotoxic, stimuli-responsive hydrogels with antibacterial activity. Biomacromolecules.

[120] Hoque J., Sangaj N., Varghese S. (2018). Stimuli-responsive supramolecular hydrogels and their applications in regenerative medicine. Macromol. Biosci..

[121] Xian S., Webber M.J. (2020). Temperature-responsive supramolecular hydrogels. J. Mater. Chem. B.

[122] Panja S., Adams D.J. (2021). Stimuli responsive dynamic transformations in supramolecular gels. Chem. Soc. Rev..

[123] Silbernagl D., Szymoniak P., Tavasolyzadeh Z., Sturm H., Topolniak I. (2024). Multiphoton lithography of interpenetrating polymer networks for tailored microstructure thermal and micromechanical properties. Small.

[124] Schwärzle D., Hou X., Prucker O., Rühe J. (2017). Polymer microstructures through two-photon crosslinking. Adv. Mat..

[125] Morozov Y.M., Quilis N.G., Diehl F., Klees S., Grün J.J., Jonas U., Dostalek J. (2022). One- and two-photon crosslinked polymer hydrogel microstructures for optical spectroscopy and biosensing applications. Proc. SPIE.

[126] Morozov Y.M., Quilis N.G., Fossati S., De Laporte L., Gusenbauer C., Weber A., Toca-Herrera J.L., Wiesner F., Jonas U., Dostalek J. (2024). Plasmon-enhanced multiphoton polymer crosslinking for selective modification of plasmonic hotspots. J. Phys. Chem. C.

[127] Zhang K., Feng Q., Fang Z., Gu L., Bian L. (2021). Structurally dynamic hydrogels for biomedical applications: Pursuing a fine balance between macroscopic stability and microscopic dynamics. Chem. Rev..

[128] Ashrafizadeh M., Tam K.C., Javadi A., Abdollahi M., Sadeghnejad S., Bahramian A. (2020). Dual physically and chemically cross-linked polyelectrolyte nanohydrogels: Compositional and pH-dependent behavior studies. Eur. Polym. J..

[129] Hwang S.M., Kim E., Wu J., Kim M.H., Lee H., Park W.H. (2024). Temperature- and pH-induced dual-crosslinked methylcellulose/chitosan-gallol conjugate composite hydrogels with improved mechanical, tissue adhesive, and hemostatic properties. Int. J. Biol. Macromol..

[130] Ansari M.J., Rajendran R.R., Mohanto S., Agarwal U., Panda K., Dhotre K., Manne R., Deepak A., Zafar A., Yasir M. (2022). Poly(N-isopropylacrylamide)-based hydrogels for biomedical applications: A review of the state-of-the-art. Gels.

[131] Luo C., Huang M., Sun X., Wei N., Shi H., Li H., Lin M., Sun J. (2022). Super-strong, nonswellable, and biocompatible hydrogels inspired by human tendons. ACS Appl. Mater. Interfaces.

[132] Li W., Guan Q., Li M., Saiz E., Hou X. (2023). Nature-inspired strategies for the synthesis of hydrogel actuators and their applications. Prog. Polym. Sci..

[133] Fan H., Gong J.P. (2020). Fabrication of bioinspired hydrogels: Challenges and opportunities. Macromolecules.

[134] Wang Z., Wei H., Huang Y., Wei Y., Chen J. (2023). Naturally sourced hydrogels: Emerging fundamental materials for next-generation healthcare sensing. Chem. Soc. Rev..

[135] Koca E.I., Bozdag G., Cayli G., Kazan D., Hatir P.C. (2020). Thermoresponsive hydrogels based on renewable resources. J. Appl. Polym. Sci..

[136] Jankau J., Błażyńska-Spychalska A., Kubiak K., Jędrzejczak-Krzepkowska M., Pankiewicz T., Ludwicka K., Dettlaff A., Pęksa R. (2022). Bacterial cellulose properties fulfilling requirements for a biomaterial of choice in reconstructive surgery and wound healing. Front. Bioeng. Biotechnol..

[137] Torres F.G., Commeaux S., Troncoso O.P. (2012). Biocompatibility of bacterial cellulose based biomaterials. J. Funct. Biomater..

[138] Rzhepakovsky I., Piskov S., Avanesyan S., Sizonenko M., Timchenko L., Anfinogenova O., Nagdalian A., Blinov A., Denisova E., Kochergin S. (2024). Composite of bacterial cellulose and gelatin: A versatile biocompatible scaffold for tissue engineering. Int. J. Biol. Macromol..

[139] Farazin A., Zhang C., Ghasemi A.H. (2023). Preparation and identification of new antibacterial and biocompatible dressings based on gelatin/polyvinyl alcohol and castor oil. J. Polym. Res..

[140] Trubetskaya A., Leppiniemi J., Lipponen S., Lombardo S., Thielemans W., Maloney T., Pääkkönen T., Kesari K.K., Ruokolainen J., Hytönen V.P. (2024). Thermoresponsive and biocompatible poly(N-isopropylacrylamide)–cellulose nanocrystals hydrogel for cell growth. Mater. Adv..

[141] Lanzalaco S., Mingot J., Torras J., Alemán C., Armelin E. (2023). Recent advances in poly(N-isopropylacrylamide) hydrogels and derivatives as promising materials for biomedical and engineering emerging applications. Adv. Engineer. Mat..

[142] Xue X., Hu Y., Deng Y., Su J. (2021). Recent advances in design of functional biocompatible hydrogels for bone tissue engineering. Adv. Funct. Mat..

[143] Sood N., Bhardwaj A., Mehta S., Mehta A. (2016). Stimuli-responsive hydrogels in drug delivery and tissue engineering. Drug Deliv..

[144] Jacob S., Nair A.B., Shah J., Sreeharsha N., Gupta S., Shinu P. (2021). Emerging role of hydrogels in drug delivery systems, tissue engineering and wound management. Pharmaceutics.

[145] Dong R., Guo B. (2021). Smart wound dressings for wound healing. Nano Today.

[146] Guo Z., Liu H., Shi Z., Lin L., Li Y., Wang M., Pan G., Lei Y., Xue L. (2022). Responsive hydrogel-based microneedle dressing for diabetic wound healing. J. Mater. Chem. B.

[147] Zhang S., Ge G., Qin Y., Li W., Dong J., Mei J., Ma R., Zhang X., Bai J., Zhu C. (2023). Recent advances in responsive hydrogels for diabetic wound healing. Materials Today Bio.

[148] Visan A.I., Negut I. (2024). Development and applications of PLGA hydrogels for sustained delivery of therapeutic agents. Gels.

[149] Hageneder S., Jungbluth V., Soldo R., Petri C., Pertiller M., Kreivi M., Weinhäusel A., Jonas U., Dostalek J. (2021). Responsive hydrogel binding matrix for dual signal amplification in fluorescence affinity biosensors and peptide microarrays. ACS Appl. Mater. Interfaces.

[150] Sun S., Chen J. (2024). Recent advances in hydrogel-based biosensors for cancer detection. ACS Appl. Mater. Interfaces.

[151] Lin X., Wang Z., Jia X., Chen R., Qin Y., Bian Y., Sheng W., Li S., Gao Z. (2023). Stimulus-responsive hydrogels: A potent tool for biosensing in food safety. Trends Food Sci..

[152] Bhat A., Amanor-Boadu J.M., Guiseppi-Elie A. (2020). Toward impedimetric measurement of acidosis with a pH-responsive hydrogel sensor. ACS Sens..

[153] Endo T., Ikeda R., Yanagida Y., Hatsuzawa T. (2008). Stimuli-responsive hydrogel–silver nanoparticles composite for development of localized surface plasmon resonance-based optical biosensor. Anal. Chim. Acta.

[154] Tokarev I., Tokareva I., Gopishetty V., Katz E., Minko S. (2010). Specific biochemical-to-optical signal transduction by responsive thin hydrogel films loaded with noble metal nanoparticles. Adv. Mat..

[155] Belger C., Weis J.G., Egap E., Swager T.M. (2015). Colorimetric stimuli-responsive hydrogel polymers for the detection of nerve agent surrogates. Macromolecules.

[156] Sun Y., Lv Y., Zhang Y., Wang Z. (2023). A stimuli-responsive colorimetric aptasensor based on the DNA hydrogel-coated MOF for fumonisin B1 determination in food samples. Food Chem..

[157] Kang T., Cha G.D., Park O.K., Cho H.R., Kim M., Lee J., Kim D., Lee B., Chu J., Koo S. (2023). Penetrative and sustained drug delivery using injectable hydrogel nanocomposites for postsurgical brain tumor treatment. ACS Nano.

[158] Lin S.-H., Huang A.P.-H., Hsu S.-H. (2023). Injectable, micellar chitosan self-healing hydrogel for asynchronous dual-drug delivery to treat stroke rats. Adv. Funct. Mat..

[159] Wang Y., Zheng C., Wu Y., Zhang B., Hu C., Guo C., Kong Q., Wang Y. (2023). An injectable and self-strengthening nanogel encapsuled hydrogel gene delivery system promotes degenerative nucleus pulposus repair. Compos. B Eng..

[160] Sheng Y., Gao J., Yin Z.-Z., Kang J., Kong Y. (2021). Dual-drug delivery system based on the hydrogels of alginate and sodium carboxymethyl cellulose for colorectal cancer treatment. Carbohydr. Polym..

[161] Tian W., Wang C., Chu R., Ge H., Sun X., Li M. (2023). Injectable hydrogel nanoarchitectonics with near-infrared controlled drug delivery for in situ photothermal/endocrine synergistic endometriosis therapy. Biomater. Res..

[162] Li J., Wang Z., Han H., Xu Z., Li S., Zhu Y., Chen Y., Ge L., Zhang Y. (2022). Short and simple peptide-based pH-sensitive hydrogel for antitumor drug delivery. Chin. Chem. Lett..

[163] Ge P., Chang S., Wang T., Zhao Q., Wang G., He B. (2023). An antioxidant and antibacterial polydopamine-modified thermo-sensitive hydrogel dressing for Staphylococcus aureus-infected wound healing. Nanoscale.

[164] Jiang Y., Zhang X., Zhang W., Wang M., Yan L., Wang K., Han L., Lu X. (2022). Infant skin friendly adhesive hydrogel patch activated at body temperature for bioelectronics securing and diabetic wound healing. ACS Nano.

[165] Yang M., He S., Su Z., Yang Z., Liang X., Wu Y. (2020). Thermosensitive injectable chitosan/collagen/β-glycerophosphate composite hydrogels for enhancing wound healing by encapsulating mesenchymal stem cell spheroids. ACS Omega.

[166] Jiang J., Ding J., Wu X., Zeng M., Tian Y., Wu K., Wei D., Sun J., Guo Z., Fan H. (2023). Flexible and temperature-responsive hydrogel dressing for real-time and remote wound healing monitoring. J. Mater. Chem. B.

[167] Mitra S., Chakraborty A.J., Tareq A.M., Emran T.B., Nainu F., Khusro A., Idris A.M., Khandaker M.U., Osman H., Alhumaydhi F.A. (2022). Impact of heavy metals on the environment and human health: Novel therapeutic insights to counter the toxicity. J. King Saud Univ. Sci..

[168] Darban Z., Shahabuddin S., Gaur R., Ahmad I., Sridewi N. (2022). Hydrogel-based adsorbent material for the effective removal of heavy metals from wastewater: A comprehensive review. Gels.

[169] Zhao Z., Zhang Z., Zhu Z., Zou X., Zhao Y., Shi J., Wang J. (2022). Photothermal responsive hydrogel for adsorbing heavy metal ions in aqueous solution. Colloids Surf. A Physicochem. Eng. Asp..

[170] Tohamy H.-A.S., El-Sakhawy M., Strachota B., Strachota A., Pavlova E., Barbosa S.M., Kamel S. (2023). Temperature- and pH-responsive super-absorbent hydrogel based on grafted cellulose and capable of heavy metal removal from aqueous solutions. Gels.

[171] Zheng L., Seidi F., Wu W., Pan Y., Xiao H. (2023). Dual-functional lignin-based hydrogels for sustained release of agrochemicals and heavy metal ion complexation. Int. J. Biol. Macromol..

[172] Dai L., Wang Y., Li Z., Wang X., Duan C., Zhao W., Xiong C., Nie S., Xu X., Ni Y. (2020). A multifunctional self-crosslinked chitosan/cationic guar gum composite hydrogel and its versatile uses in phosphate-containing water treatment and energy storage. Carbohydr. Polym..

[173] He T., Yang Y., Chen X.-B. (2023). Preparation, stimulus–response mechanisms and applications of micro/nanorobots. Micromachines.

[174] Ren Z., Hu W., Dong X., Sitti M. (2019). Multi-functional soft-bodied jellyfish-like swimming. Nat. Commun..

[175] Lee Y., Song W.J., Sun J.-Y. (2020). Hydrogel soft robotics. Mater. Today Phys..

[176] Shen Z., Chen F., Zhu X., Yong K.-T., Gu G. (2020). Stimuli-responsive functional materials for soft robotics. J. Mater. Chem. B.

[177] Zhao T., Tan Y., Li Y., Wang X. (2025). Ionic fuel-powered hydrogel actuators for soft robotics. J. Colloid. Interface Sci..

[178] Liu H., Chu H., Yuan H., Li D., Deng W., Fu Z., Liu R., Liu Y., Han Y., Wang Y. (2024). Bioinspired multifunctional self-sensing actuated gradient hydrogel for soft-hard robot remote interaction. Nano-Micro Lett..

[179] Siebenmorgen C., Wang C., Navarro L.B., Parisi D., Misra S., Venkiteswaran V.K., van Rijn P. (2024). Minimally designed thermo-magnetic dual responsive soft robots for complex applications. J. Mater. Chem. B.

[180] López-Díaz A., Martín-Pacheco A., Rodríguez A.M., Herrero M.A., Vázquez A.S., Vázquez E. (2020). Concentration gradient-based soft robotics: Hydrogels out of water. Adv. Funct. Mat..

[181] López-Díaz A., Vázquez A.S., Vázquez E. (2024). Hydrogels in soft robotics: Past, present, and future. ACS Nano.

[182] Diehl F., Hageneder S., Fossati S., Auer S.K., Dostalek J., Jonas U. (2022). Plasmonic nanomaterials with responsive polymer hydrogels for sensing and actuation. Chem. Soc. Rev..

[183] Molina M., Asadian-Birjand M., Balach J., Bergueiro J., Miceli E., Calderón M. (2015). Stimuli-responsive nanogel composites and their application in nanomedicine. Chem. Soc. Rev..

[184] Jeon S.-J., Hauser A.W., Hayward R.C. (2017). Shape-morphing materials from stimuli-responsive hydrogel hybrids. Acc. Chem. Res..

[185] Michalicha A., Belcarz A., Giannakoudakis D.A., Staniszewska M., Barczak M. (2024). Designing composite stimuli-responsive hydrogels for wound healing applications: The state-of-the-art and recent discoveries. Materials.

[186] Rana M.M., De la Hoz Siegler H. (2024). Evolution of hybrid hydrogels: Next-generation biomaterials for drug delivery and tissue engineering. Gels.

[187] Mohan A., Santhamoorthy M., Lee Y.C. (2024). Recent advances in the pH-responsive organic–inorganic mesoporous hybrid silica for targeted drug delivery. Eur. Polym. J..

[188] Lee W., Kim D., Lee S., Park J., Oh S., Kim G., Lim J., Kim J. (2018). Stimuli-responsive switchable organic-inorganic nanocomposite materials. Nano Today.

[189] Camerin F., Fernández-Rodríguez M.Á., Rovigatti L., Antonopoulou M.-N., Gnan N., Ninarello A., Isa L., Zaccarelli E. (2019). Microgels adsorbed at liquid–liquid interfaces: A joint numerical and experimental study. ACS Nano.

[190] Mourran A., Zhang H., Vinokur R., Möller M. (2017). Soft microrobots employing nonequilibrium actuation via plasmonic heating. Adv. Mat..

[191] Kannan M.R.R., Tata B.V.R., Dasgupta R., Ahlawat S., Gupta P.K. (2011). Optical trapping of thermo-responsive microgel particles by holographic optical tweezers. AIP Conf. Proc..

[192] Gupta D.K., Karthickeyan D., Tata B.V.R., Ravindran T.R. (2016). Temperature-driven volume phase transition of a single stimuli-responsive microgel particle using optical tweezers. Colloid. Polym. Sci..

[193] Karthickeyan D., Gupta D.K., Tata B.V.R. (2016). Identification of volume phase transition of a single microgel particle using optical tweezers. J. Opt..

[194] Auer S.K., Fossati S., Morozov Y., Mor D.C., Jonas U., Dostalek J. (2022). Rapid actuation of thermo-responsive polymer networks: Investigation of the transition kinetics. J. Phys. Chem. B.

[195] Militaru A., Lasanta A., Frimmer M., Bonilla L.L., Novotny L., Rica R.A. (2021). Kovacs memory effect with an optically levitated nanoparticle. Phys. Rev. Lett..

